# An improved advertising CTR prediction approach based on the fuzzy deep neural network

**DOI:** 10.1371/journal.pone.0190831

**Published:** 2018-05-04

**Authors:** Zilong Jiang, Shu Gao, Mingjiang Li

**Affiliations:** 1 School of Computer Science and Technology, Wuhan University of Technology, Wuhan, Hubei Province, China; 2 School of Computer and Information, Qiannan Normal University for Nationalities, Duyun, Guizhou Province, China; Southwest University, CHINA

## Abstract

Combining a deep neural network with fuzzy theory, this paper proposes an advertising click-through rate (CTR) prediction approach based on a fuzzy deep neural network (FDNN). In this approach, fuzzy Gaussian-Bernoulli restricted Boltzmann machine (FGBRBM) is first applied to input raw data from advertising datasets. Next, fuzzy restricted Boltzmann machine (FRBM) is used to construct the fuzzy deep belief network (FDBN) with the unsupervised method layer by layer. Finally, fuzzy logistic regression (FLR) is utilized for modeling the CTR. The experimental results show that the proposed FDNN model outperforms several baseline models in terms of both data representation capability and robustness in advertising click log datasets with noise.

## Introduction

Internet advertising is regarded as an effective advertising communication approach due to its strong targeted communication ability. Therefore, increasing numbers of researchers from industry and academia have investigated internet advertising, and it has become an important source of income for internet companies.

The cost per click (CPC) model [1] is one of the most common payment models in internet advertising. Approximately 66% of advertising transactions depend on the CPC because the CPC can more accurately reveal the conversion rate compared with the other models [2]. In the CPC model, the click-through rate (CTR) is a significant index for measuring the effect of advertisement placement.

To address this task, Chapelle O. et al. propose a machine learning framework that uses Maximum Entropy to implement a logistic regression model and can address billions of samples and hundreds of millions of parameters. A two-phase feature selection algorithm is provided to reduce the need for domain expertise: a generalized mutual information method is used to select the feature groups that are used in the model; next, feature hashing is used to regulate the size of the models [3]. McMahan et al. adopted a logistic regression model to solve advertising CTR problems for Google. Using multiple characteristics, including user information, search keywords, advertising data, and relative metadata, with advertisements as the input of the model, these researchers proposed an online sparse learning algorithm to the train model [4]. These above methods are recently used to address CTR prediction through the logistic regression (LR) model in industry. These methods can appropriately address a large number of features in real advertising system in industry, and can be efficiently parallelized in a distributed computing system. However, they cannot learn complex mapping relations from data because of their limited data representation capability.

T. A. Anh-Phuong presented an online learning algorithm for click-through rate prediction, known as Follow The Regularized Factorized Leader (FTRFL), which combines the Follow-The-Regularized-Leader Proximal (FTRL-P) algorithm with per-coordinate learning rates to obtain factorization machines. These researchers attempt to obtain both the sparsity provided by FTRL-Proximal and the ability to estimate higher-order features based on FM [5]. Z. Pan et al. proposed a Sparse Factorization Machine (SFM) model to solve the problem of the sparsity of the ad transaction dataset. In this model, the Laplace distribution is introduced to model the parameters because it can better fit the sparse data, which has a high ratio of zero elements. Furthermore, these researchers parallelized the SFM model on the Spark platform to support CTR prediction in a real advertising system [2]. These above methods are recently adopted to address CTR prediction through Factorization Machines (FMs) in academia. These methods can alleviate this problem of feature learning by compressing the sparse features into a dense vector space and using the second-order feature interactions. However, when facing a dataset with complex relations, they cannot sufficiently capture the higher-order non-linear representative features and complex inherent mapping relations among the users, contexts and ads in the advertising datasets.

A. I. Baqapuri and I. Trofimov adopted Artificial Neural Networks (ANNs) to predict the advertising CTR and obtained a better effect than that of the logistic regression model [6]. Dave and other researchers proposed a model that inherits the click information of rare/new ads from frequent ads that are semantically related to calculate the CTR values for newly created ads or rare ads that do not have sufficient historical information. The semantic features are derived from the search ad click-through graphs and advertiser account information. Next, gradient boosted decision trees (GBDTs) is adopted to learn from these similarity features. Experiments demonstrate that this learned model, using these features, obtains good CTR prediction performance for new ads [7]. Y Juan et al. first used trees in GBDT to generate abstract nonlinear features and then combined the original features as input features, which were fed into the FM model for the advertising prediction [8]. These above methods are recently used to address CTR prediction through models ensemble technique, and they give state-of-the-art ideas for industry and academia.

Zhang and other researchers used recurrent neural networks (RNNs) to predict the CTR of sponsored search advertising. Using back-propagation through time (BPTT) to train their model, these researchers obtained more accurate results for advertising CTR prediction than neural networks and logistic regression models [9]. Yu adopted the improved regression neural network to predict advertising CTR by using long short-term memory (LSTM) to modify RNN. This method effectively prevents the explosion or vanishing of the gradient [10]. Jiang et al. adopted the DBNLR model, which integrates a deep belief network (DBN) with logistic regression (LR) to address the problem of CTR prediction. A DBN stacked of RBMs is used to obtain abstract features from original advertisement datasets, and then a regression model is adopted to calculate the CTR prediction value [11]. Junxuan Chen et al. proposed a novel deep neural network that contains convolution layers to extract representative visual features, and fully connected layers learn the complex and effective nonlinear features based on the basic visual features. Finally, these features are fed into a logistic regression model to predict the CTR value [12]. These above methods are state-of-the-art solutions which are adopted to address CTR prediction task through deep learning technology. In these above methods, a deep architecture model is usually used to automatically extract the features of advertising click log data, and a regression model is subsequently trained to model the advertising CTR. Because a deep architecture is used to learn the features, there is no need for these methods to depend on prior knowledge or human labor; they can effectively fit complex nonlinear mapping relations in advertising datasets. However, when the dataset contains noisy data such as missing values and outliers, these methods may exhibit performance degradation.

A common strategy for dealing with this problem is to identify and delete records with outliers during data preprocessing. However, deleting entire records results in the loss of much valuable and clean information. The other common strategy is to replace missing values and outliers with “0” or “NULL”; however, the records with outliers are preserved in the training set, which also affects the accuracy of CTR prediction.

Uncertainties not only exist in the data themselves but occur at each phase of big data processing. For instance, the collected data may be created by faulty sensors or provided by not fully informed customers; the outputs of specific artificial intelligent algorithms also contain uncertainties. In these cases, fuzzy set techniques could be one of the most efficient tools to handle various types of uncertainties [13]. Because the parameters of these above deep architecture models are constants, and the learning processes of the parameters are constrained in a relatively small space, these methods have insufficient representation capability and relatively weak robustness when the training data have been interrupted by uncertainties. Meanwhile, fuzzy set techniques would be more efficient if they are used associated with other decision making techniques, such as probability, neural networks, etc., because each type of techniques exhibit their own strengths of representing and handling information granularity [13].

Due to the lack of a comprehensive understanding of the advertising datasets with noises, it is often difficult for these methods to ensure that the extracted features capture the optimal information for predicting the CTR. These deficiencies affect the accuracy and fitness of these methods. According to the advantages of fuzzy set techniques, this paper proposes a novel CTR prediction method based on the fuzzy deep neural network (FDNN) to address advertising datasets with noise.

The main contributions of this paper can be summarized as follows:

1. This paper proposes an FDNN model in which FDBN is used to automatically extract abstract and complicated features from raw advertising datasets without any artificial intervention or prior knowledge and subsequently uses fuzzy logistic regression to model the CTR.

2. Fuzzy set techniques are introduced into the Gaussian-Bernoulli Restricted Boltzmann Machine (GBRBM), Restricted Boltzmann Machine (RBM) and logistic regression models to construct basic components of FDNN, and corresponding learning algorithms are presented in detail.

3. We conduct extensive experiments on real-world datasets to demonstrate the effectiveness and efficiency of the proposed FDNN model. The impacts of several baseline models are also discussed. Experiments results show the superiority of the proposed method. On the first dataset, the results show that the proposed method achieves competitive performances compared with the state-of-the-art models in terms of both data representation capability and robustness.

## Materials and methods

### Relative theories of fuzzy numbers and fuzzy functions

#### Fuzzy number

**Definition 1**. Triangular Fuzzy Number

A fuzzy number $\bar{\theta }$ (fuzzy set) on a real domain can be defined as a triangular fuzzy number if its membership function $\mu_{\bar{\theta }}\left(x\right):R\to \left[0,1\right]$ is equal to [14], and it is shown in S1 Fig.

$$\begin{array}{c} \begin{array}{c} \mu_{\bar{\theta }}\left(x\right)=\bar{\theta }\left(x\right)=\{\begin{array}{c} \frac{x}{m-l}-\frac{l}{m-l},x\in \left[l,m\right]\\ \frac{x}{m-u}-\frac{u}{m-u},x\in \left[m,u\right]\\ 0,otherwise \end{array}, \end{array} \end{array}$$ (1)

where *l* ≤ *m* ≤ *u*, *l* and *u* stand for the lower and upper values of the support of fuzzy number $\bar{\theta }$ (fuzzy set), respectively; and *m* is the most likely value (middle value), that is, when *x* = *m*, *x* belongs to $\bar{\theta }$. The triangular fuzzy number can be denoted by (*l*, *m*, *u*). The support of $\bar{\theta }$ is the set of elements {*x* ∈ *R*|*l* < *x* < *u*}. When *l* = *m* = *u*, it is a non-fuzzy number.

**Definition 2**. *α*-cuts of a fuzzy set

The *α*-cut of fuzzy set $\bar{\theta }$, represented by $\bar{\theta }\left[\alpha \right]$, is defined as $\bar{\theta }\left[\alpha \right]=\left\{x\in \Omega |\bar{\theta }\geq \alpha \right\}=\left[\theta_{L},\theta_{R}\right]$, where 0 < *α* ≤ 1, *θ*_*L*_ is the lower value of $\bar{\theta }\left[\alpha \right]$ and *θ*_*R*_ is the upper value of $\bar{\theta }\left[\alpha \right]$ [15, 16].

#### Fuzzy function

**Definition 3**. Fuzzy Function

Extended from a real-valued function *f*: *Y* = *f*(*x*, ***θ***), the fuzzy function $\bar{f}$ can be defined as [16]

$$\begin{array}{c} \begin{array}{c} \bar{Y}=\bar{f}\left(x,\bar{\theta }\right), \end{array} \end{array}$$ (2)

where ***θ*** and $\bar{\theta }$ are parameters of functions *f* and $\bar{f}$ [16, 17], $\bar{Y}$ is the fuzzy output set.

The membership function $\bar{Y}\left(y\right)$ can be expressed as

$$\begin{array}{c} \begin{array}{c} \bar{Y}\left(y\right)=sup_{\theta }\left\{min\left({\bar{\theta }}_{1}\left(\theta_{1}\right),...,{\bar{\theta }}_{n}\left(\theta_{n}\right)\right)|f\left(x,\theta \right)=y\right\}, \end{array} \end{array}$$ (3)

where ***θ*** = (*θ*_1_, …, *θ*_*n*_)^*T*^, and $\bar{\theta }={\left({\bar{\theta }}_{1},...,{\bar{\theta }}_{n}\right)}^{T}$

**Property 1**. Real Domain Interval Arithmetic

For two real domain intervals [*a*, *b*] and [*c*, *d*], the interval arithmetic can be defined as [16, 18]

[*a*, *b*] + [*c*, *d*] = [*a* + *c*, *b* + *d*];

[*a*, *b*] − [*c*, *d*] = [*a* − *c*, *b* − *d*];

[*a*, *b*] × [*c*, *d*] = [*min*(*a* × *c*, *a* × *d*, *b* × *c*, *b* × *d*), *max*(*a* × *c*, *a* × *d*, *b* × *c*, *b* × *d*)];

[*a*, *b*] ÷ [*c*, *d*] = [*min*(*a* ÷ *c*, *a* ÷ *d*, *b* ÷ *c*, *b* ÷ *d*), *max*(*a* ÷ *c*, *a* ÷ *d*, *b* ÷ *c*, *b* ÷ *d*)].

**Definition 4**. *α*-Cuts of a Fuzzy Function

*α*-Cuts of $\bar{Y}$: For a continuous function *f*, the *α*-Cuts of $\bar{Y}$, namely $\bar{Y}\left[\alpha \right]=\left[{\bar{Y}}_{1}\left[\alpha \right],{\bar{Y}}_{2}\left[\alpha \right]\right]$, can be expressed as [16]

$$\begin{array}{c} \begin{array}{c} \left\{ \begin{array}{c} {\bar{Y}}_{1}\left[\alpha \right]=min\left\{Y\left(\theta ,x\right)|\theta \in \bar{\theta }\left[\alpha \right]\right\}\\ {\bar{Y}}_{2}\left[\alpha \right]=max\left\{Y\left(\theta ,x\right)|\theta \in \bar{\theta }\left[\alpha \right]\right\} \end{array}.\right. \end{array} \end{array}$$ (4)

It is infeasible to calculate the membership function $\bar{Y}\left(y\right)$ using formulas (2) and (3) because this requires maximization and minimization of the original function. This paper adopts *α*-cuts and interval arithmetic to solve this problem.

$$\begin{array}{c} \begin{array}{c} \bar{Y}\left[\alpha \right]=f\left(x,\bar{\theta }\left[\alpha \right]\right) \end{array} \end{array}$$ (5)

The membership function $\bar{Y}\left(y\right)$ of a fuzzy function can be obtained by interval arithmetic because intervals $\bar{\theta }\left[\alpha \right]$ are easy to calculate. However, when *f* is very complex, interval arithmetic will be NP-hard; therefore, this paper introduces a defuzzification approach to handle this problem.

#### Defuzzification of the fuzzy function

The centroid approximate solution [19] is employed to defuzzify the fuzzy function $\bar{Y}=\bar{f}\left(x,\bar{\theta }\right)$ in this paper.

The centroid of fuzzy function $\bar{f}\left(x,\bar{\theta }\right)$ can be denoted by $f_{c}\left(x,\bar{\theta }\right)$ [19]:

$$\begin{array}{c} \begin{array}{c} f_{c}\left(x,\bar{\theta }\right)=\frac{\int \theta f(x,\theta )d\theta }{\int f(x,\theta )d\theta },\theta \in \bar{\theta }. \end{array} \end{array}$$ (6)

However, directly calculating formula (6) is difficult because it involves integrals. The centroid can be approximated through many *α*-cuts of the fuzzy function in discrete form. $\bar{\theta }$ is a vector of fuzzy numbers and an *α*-cut of $\bar{\theta }$ can be denoted as $\bar{\theta }\left[\alpha \right]=\left[\theta_{L},\theta_{R}\right]$, where ***θ***_*L*_ and ***θ***_*R*_ are lower and upper bounds, respectively, of the interval with respect to *α*. When *x* is nonnegative, $\bar{f}\left(x,\bar{\theta }\right)$ is a monotonically decreasing function with respect to parameters $\bar{\theta }$. Thus, according to the interval arithmetic principle, definition 3 and formula (5), the *α*-cut of $\bar{f}\left(x,\bar{\theta }\right)$ is given by

$$\begin{array}{c} \begin{array}{c} \bar{f}\left(x,\bar{\theta }\right)\left[\alpha \right]=f\left(x,\bar{\theta }\left[\alpha \right]\right)=\left[f\left(x,\theta_{L}\right),f\left(x,\theta_{R}\right)\right]. \end{array} \end{array}$$ (7)

The approximate centroid of fuzzy function $\bar{f}\left(x,\bar{\theta }\right)$ can be obtained by [20]

$$\begin{array}{c} \begin{array}{c} f_{c}\left(x,\bar{\theta }\right)\approx \frac{\sum_{i=1}^{M}\alpha_{i}\left[f\left(x,\theta_{iL}\right),f\left(x,\theta_{iR}\right)\right]}{2\sum_{i=1}^{M}\alpha_{i}}, \end{array} \end{array}$$ (8)

where ***α*** = (*α*_1_, …, *α*_*N*_), ***α*** ∈ [0, 1]^*N*^ and $\bar{\theta }\left[\alpha_{i}\right]=\left[\theta_{iL},\theta_{iR}\right]$.

Although all the *α*-cutsare bounded intervals, this paper takes only the special case where *α*_*i*_ = 1 into consideration. Let ***θ*** = [***θ***_*L*_, ***θ***_*R*_]. Formula (8) can be expressed as [20]

$$\begin{array}{c} \begin{array}{c} f_{c}\left(x,\bar{\theta }\right)\approx \frac{1}{2}\left[f\left(x,\theta_{L}\right)+f\left(x,\theta_{R}\right)\right]. \end{array} \end{array}$$ (9)

Therefore, fuzzy function $\bar{Y}$ can be expressed as

$$\begin{array}{c} \begin{array}{c} \bar{Y}=\bar{f}\left(x,\bar{\theta }\right)=f_{c}\left(x,\bar{\theta }\right)\approx \frac{1}{2}\left[f\left(x,\theta_{L}\right)+f\left(x,\theta_{R}\right)\right]. \end{array} \end{array}$$ (10)

### Advertising CTR prediction based on the fuzzy deep neural network

#### Basic fuzzy components and corresponding learning algorithms

Inspired by the idea of above fuzzy theory and methods [16–20], we first provide a detailed introduction and describe the corresponding learning algorithms of several basic components that have been modified using fuzzy technology.

*A*. *FRBM and its Learning Algorithm*

Fuzzy restricted Boltzmann machine (FRBM) is a symmetric neural network with binary nodes that is based on an energy model. It contains a set of visual binary nodes ***v*** ∈ {0, 1}^*D*^ and another set of hidden binary nodes ***h*** ∈ {0, 1}^*F*^. There are no connections between different nodes of the same hidden layer or between different nodes of the same visual layer. Fuzzy parameters are employed to govern the FRBM model. The structure of FRBM is described in S2 Fig.

The fuzzy energy function of FRBM can be expressed as [20]

$$\begin{array}{c} \begin{array}{c} \bar{E}\left(x,h;\bar{\theta }\right)=-{\bar{b}}^{T}x-{\bar{c}}^{T}h-h^{T}\bar{W}x. \end{array} \end{array}$$ (11)

In this formula, $\bar{\theta }=\left\{\bar{b},\bar{c},\bar{W}\right\}$, $\bar{b}$ and $\bar{c}$ are the offsets, and $\bar{W}$ is the connection weight between the *i*th visible node and the *j*th hidden node.

According to the free energy function $F\left(x,\theta \right)=-log\sum_{\tilde{h}}e^{-E(x,\tilde{h},\theta )}$, the fuzzy free energy function $\bar{F}$ is expressed as

$$\begin{array}{c} \begin{array}{c} \bar{F}\left(x,\bar{\theta }\right)=-log\sum_{\tilde{h}}e^{-\bar{E}\left(x,\tilde{h},\bar{\theta }\right)}. \end{array} \end{array}$$ (12)

If $\bar{F}\left(x,\bar{\theta }\right)$ is used directly to define the probability, it is difficult to calculate the fuzzy probability, fuzzy maximum likelihood and fuzzy objective function in the optimization. Therefore, $\bar{F}\left(x,\bar{\theta }\right)$ needs to be defuzzifized to transform the optimization into regular maximum-likelihood optimization. This paper adopts a centroid method to defuzzify $\bar{F}\left(x,\bar{\theta }\right)$.

The centroid of $\bar{F}\left(x,\bar{\theta }\right)$ is expressed as $F_{c}\left(x,\bar{\theta }\right)$, and the probability can be defined as

$$\begin{array}{c} \begin{array}{c} P_{c}\left(x,\bar{\theta }\right)=\frac{e^{-F_{c}\left(x,\bar{\theta }\right)}}{Z},Z=\sum_{\tilde{x}}e^{-F_{c}\left(\tilde{x},\bar{\theta }\right)}. \end{array} \end{array}$$ (13)

This paper selects the negative log-likelihood as the objective function of FRBM:

$$\begin{array}{c} \begin{array}{c} L\left(\bar{\theta },D\right)=-\sum_{x\in D}logP_{c}\left(x,\bar{\theta }\right). \end{array} \end{array}$$ (14)

In the formula, *D* denotes the training dataset.

The learning algorithm of FRBM finds the parameters $\bar{\theta }$ that minimize the objective function $L(\bar{\theta },D)$ ($minL_{\bar{\theta }}\left(\bar{\theta },D\right)$). The paper adopts the centroid approximation method to defuzzify $\bar{F}\left(x,\bar{\theta }\right)$:

$$\begin{array}{c} \begin{array}{c} \bar{F}\left(x,\bar{\theta }\right)=F_{c}\left(x,\bar{\theta }\right)\approx \frac{1}{2}\left[F\left(x,\theta_{L}\right)+F\left(x,\theta_{R}\right)\right]. \end{array} \end{array}$$ (15)

The gradients of $L(\bar{\theta },D)$ with respect to ***θ***_*L*_ can be expressed as

$$\begin{array}{c} \begin{array}{c} -\frac{\partial logP_{c}\left(x,\bar{\theta }\right)}{\partial \theta_{L}}=\frac{\partial F_{c}\left(x,\theta_{L}\right)}{\partial \theta_{L}}-E_{p}[\frac{\partial F_{c}\left(x,\theta_{L}\right)}{\partial \theta_{L}}]. \end{array} \end{array}$$ (16)

In the formula, *E*_*p*_(⋅) is the expectation over the target probability distribution *P*.

Correspondingly,

$$\begin{array}{c} \begin{array}{c} -\frac{\partial logP_{c}\left(x,\bar{\theta }\right)}{\partial \theta_{R}}=\frac{\partial F_{c}\left(x,\theta_{R}\right)}{\partial \theta_{R}}-E_{p}[\frac{\partial F_{c}\left(x,\theta_{R}\right)}{\partial \theta_{R}}]. \end{array} \end{array}$$ (17)

Because it is difficult to calculate the expectation $E_{p}[\frac{\partial F_{c}\left(x,\theta \right)}{\partial \theta }]$, an approximation approach is employed. After defuzzifying the objective function, Gibbs sampling [21] is adopted to sample from these conditional distributions.

For FRBM, the fuzzy conditional probabilities can be expressed as $\bar{P}\left(h_{j}=1|x\right)=\sigma \left({\bar{c}}_{j}+{\bar{W}}_{\cdot j}x\right)$ and $\bar{P}\left(x_{i}=1|h\right)=\sigma \left({\bar{b}}_{i}+{\bar{W}}_{i\cdot }^{T}h\right)$. The *α*-cuts of the fuzzy conditional probabilities are described as $\bar{P}\left(h_{j}=1|x\right)\left[\alpha \right]=\left[P_{L}\left(h_{j}=1|x\right),P_{R}\left(h_{j}=1|x\right)\right]$ and $\bar{P}\left(x_{i}=1|h\right)\left[\alpha \right]=\left[P_{L}\left(x_{i}=1|h\right),P_{R}\left(x_{i}=1|h\right)\right]$.

In these formulas, *P*_*L*_(*h*_*j*_|***x***), *P*_*R*_(*h*_*j*_|***x***), *P*_*L*_(*x*_*i*_|***h***) and *P*_*R*_(*x*_*i*_|***h***) are the conditional probabilities with respect to the lower bounds and upper bounds of the parameters. Consequently,

$$\begin{array}{c} \begin{array}{c} P_{L}\left(h_{j}|x\right)=P\left(h_{j}|x;\theta_{L}\right)=\sigma \left(c_{j}^{L}+W_{\cdot j}^{L}x\right),\\ P_{R}\left(h_{j}|x\right)=P\left(h_{j}|x;\theta_{R}\right)=\sigma \left(c_{j}^{R}+W_{\cdot j}^{R}x\right), \end{array} \end{array}$$ (18)

and

$$\begin{array}{c} \begin{array}{c} P_{L}\left(x_{i}|h\right)=P\left(x_{i}|h;\theta_{L}\right)=\sigma \left(b_{i}^{L}+W_{i\cdot }^{L}h\right),\\ P_{R}\left(x_{i}|h\right)=P\left(x_{i}|h;\theta_{R}\right)=\sigma \left(b_{i}^{R}+W_{i\cdot }^{R}h\right). \end{array} \end{array}$$ (19)

In these formulas, $W_{ij}^{L}$ and $W_{ij}^{R}$ are the lower bound and upper bound of the connection weight, $b_{i}^{L}$ and $b_{i}^{R}$ are the lower bound and upper bound of the visible bias, and $c_{j}^{L}$ and $c_{j}^{R}$ are the lower bound and upper bound of the hidden bias [20].

According to (15), (16), (17) and the expectation estimation method of [20, 22], the gradients of $L(\bar{\theta },D)$ with respect to the fuzzy parameters of FRBM can be obtained as follows:

$$\begin{array}{c} \begin{array}{c} -\frac{\partial logP_{c}\left(x\right)}{\partial W_{ij}^{L}}=E_{p}\left[P_{L}\left(h_{j}|x\right)\cdot x_{i}^{L}\right]-P_{L}\left(h_{j}|x\right)\cdot x_{i}^{L}, \end{array} \end{array}$$

$$\begin{array}{c} \begin{array}{c} -\frac{\partial logP_{c}\left(x\right)}{\partial c_{j}^{L}}=E_{p}\left[P_{L}\left(h_{j}|x\right)\right]-P_{L}\left(h_{j}|x\right), \end{array} \end{array}$$

$$\begin{array}{c} \begin{array}{c} -\frac{\partial logP_{c}\left(x\right)}{\partial b_{i}^{L}}=E_{p}\left[P_{L}\left(x_{i}|h\right)\right]-x_{i}^{L}, \end{array} \end{array}$$

$$\begin{array}{c} \begin{array}{c} -\frac{\partial logP_{c}\left(x\right)}{\partial W_{ij}^{R}}=E_{p}\left[P_{R}\left(h_{j}|x\right)\cdot x_{i}^{R}\right]-P_{R}\left(h_{j}|x\right)\cdot x_{i}^{R}, \end{array} \end{array}$$

$$\begin{array}{c} \begin{array}{c} -\frac{\partial logP_{c}\left(x\right)}{\partial c_{j}^{R}}=E_{p}\left[P_{R}\left(h_{j}|x\right)\right]-P_{R}\left(h_{j}|x\right), \end{array} \end{array}$$

$$\begin{array}{c} \begin{array}{c} -\frac{\partial logP_{c}\left(x\right)}{\partial b_{i}^{R}}=E_{p}\left[P_{R}\left(x_{i}|h\right)\right]-x_{i}^{R}, \end{array} \end{array}$$

where *P*_*c*_(***x***) is the centroid probability.

The CD1 algorithm [22] is employed to obtain the updating rules for parameters (*θ*_*L*_ and *θ*_*R*_)to approximate the expectation $E_{p}[\frac{\partial F_{c}\left(x,\theta \right)}{\partial \theta }]$ [20, 22]. The above description is summarized as Algorithm 1.

**Algorithm 1** The learning algorithm of FRBM

**Input**: ***x***^(0)^ is an input sample of the training set;

  *ε* is the learning rate;

  ***W***^*L*^ and ***W***^*R*^ are the lower and upper bounds of connection weight matrices of the visible and hidden layers;

  ***b***^*L*^ and ***b***^*R*^ are the lower and upper bounds of bias vectors of the visible nodes;

  ***c***^*L*^ and ***c***^*R*^ are the lower and upper bounds of bias vectors of the hidden nodes.

**Output**: The latest parameters of FRBM: ***W***^*L*^, ***W***^*R*^, ***b***^*L*^, ***b***^*R*^, ***c***^*L*^, ***c***^*R*^.

 BEGIN

 1. For all hidden nodes j do

  obtain $P_{L}\left(h_{j}^{L(0)}=1|x^{\left(0\right)}\right)$ and $P_{R}\left(h_{j}^{R(0)}=1|x^{\left(0\right)}\right)$ by means of formula (18);

  sample $h_{j}^{L(0)}\in \left\{0,1\right\}$ from $P_{L}\left(h_{j}^{L(0)}|x^{\left(0\right)}\right)$;

  sample $h_{j}^{R(0)}\in \left\{0,1\right\}$ from $P_{R}\left(h_{j}^{R(0)}|x^{\left(0\right)}\right)$;

  end for

 2. For all visible nodes i do

  gain $P_{L}\left(x_{i}^{L(1)}=1|h^{L(0)}\right)$ and $P_{R}\left(x_{i}^{R(1)}=1|h^{R(0)}\right)$ by means of formula (19);

  sample $x_{i}^{L(1)}\in \left\{0,1\right\}$ from $P_{L}\left(x_{i}^{L(1)}|h^{L(0)}\right)$;

  sample $x_{i}^{R(1)}\in \left\{0,1\right\}$ from $P_{R}\left(x_{i}^{R(1)}|h^{R(0)}\right)$;

  end for

 3. For all hidden nodes j do

  obtain $P_{L}\left(h_{j}^{L(1)}=1|x^{L(1)}\right)$ and $P_{R}\left(h_{j}^{R(1)}=1|x^{R(1)}\right)$ by means of formula (18);

  sample $h_{j}^{L(1)}\in \left\{0,1\right\}$ from $P_{L}\left(h_{j}^{L(1)}|x^{L(1)}\right)$;

  sample $h_{j}^{R(1)}\in \left\{0,1\right\}$ from $P_{R}\left(h_{j}^{R(1)}|x^{R(1)}\right)$;

  end for

 4. The parameters can be updated according to the following rules:

  ***W***^*L*^ = ***W***^*L*^ + *ε*(***x***^(0)^ ⋅ *P*_*L*_(***h***^*L*(0)^ = 1|***x***^(0)^) − ***x***^*L*(1)^ ⋅ *P*_*L*_(***h***^*L*(1)^ = 1|***x***^*L*(1)^));

  ***b***^*L*^ = ***b***^*L*^ + *ε*(***x***^(0)^ − ***x***^*L*(1)^)

  ***c***^*L*^ = ***c***^*L*^ + *ε*(*P*_*L*_(***h***^*L*(0)^ = 1|***x***^(0)^ − *P*_*L*_(***h***^*L*(1)^ = 1|***x***^*L*(1)^));

  ***W***^*R*^ = ***W***^*R*^ + *ε*(***x***^(0)^ ⋅ *P*_*R*_(***h***^*R*(0)^ = 1|***x***^(0)^) − ***x***^*R*(1)^ ⋅ *P*_*R*_(***h***^*R*(1)^ = 1|***x***^*R*(1)^));

  ***b***^*R*^ = ***b***^*R*^ + *ε*(***x***^(0)^ − ***x***^*R*(1)^);

  ***c***^*R*^ = ***c***^*R*^ + *ε*(*P*_*R*_(***h***^*R*(0)^ = 1|***x***^(0)^ − *P*_*R*_(***h***^*R*(1)^ = 1|***x***^*R*(1)^));

 5. return ***W***^*L*^, ***W***^*R*^, ***b***^*L*^, ***b***^*R*^, ***c***^*L*^, ***c***^*R*^.

 END

*B*. *FGBRBM and Its Learning Algorithm*

The nodes of the visible and hidden layers in the fuzzy Gaussian-Bernoulli restricted Boltzmann machine (FGBRBM) model correspond to the Gaussian-Bernoulli nodes. In other words, the nodes of visible layer ***v*** ∈ *R*^*D*^ are Gaussian nodes and the nodes of the first hidden layer ***h*** ∈ {0, 1}^*F*^ are Bernoulli nodes (binary nodes) [23]. Their structures are described in S3 Fig. The fuzzy energy function of FGBRBM can be defined as

$$\begin{array}{c} \bar{E}\left(v,h;\bar{\theta }\right)=-{\left(v^{T}\cdot \frac{1}{\sigma }\right)}^{T}\bar{W}h-\frac{1}{2}{\left(v^{T}\cdot \frac{1}{\sigma }-{\bar{b}}^{T}\cdot \frac{1}{\sigma }\right)}^{2}-{\bar{b}}^{T}h, \end{array}$$ (20)

where *σ*_*i*_ denotes the standard deviation of *v*_*i*_ in the *i*th dimension of the Gaussian visual node.

Correspondingly, as for FGBRBM, the fuzzy conditional probability distribution for obtaining nodes of the hidden layer through nodes of the visual layer can be described as:

$$\begin{array}{c} \begin{array}{c} \bar{P}\left(h_{j}=1|v\right)=g({\bar{c}}_{j}+{\bar{W}}_{i\cdot })=g({\bar{c}}_{j}+\sum_{i}\frac{v_{i}}{\sigma_{i}}{\bar{W}}_{ij}), \end{array} \end{array}$$ (21)

where *g*(⋅) denotes the *sigmoid* function.

The formula for constructing the nodes of the visual layer through nodes of the hidden layer can be expressed as

$$\begin{array}{c} \begin{array}{c} v_{i}=N({\bar{b}}_{i}+\sigma_{i}\sum_{j=1}^{F}{\bar{W}}_{ij}h_{j},\sigma_{i}^{2}). \end{array} \end{array}$$ (22)

In this formula, *N*(*μ*, *σ*^2^) is a Gaussian probability density function, where *μ* is the mean value and *σ*^2^ denotes the variance.

The *α*-cuts of fuzzy conditional probabilities can be expressed as $\bar{P}\left(h_{j}=1|v\right)\left[\alpha \right]=\left[P_{L}\left(h_{j}=1|v\right),P_{R}\left(h_{j}=1|v\right)\right]$, ${\bar{v}}_{i}\left[\alpha \right]=N({\bar{b}}_{i}+\sigma_{i}\sum_{j=1}^{F}{\bar{W}}_{ij}h_{j},\sigma_{i}^{2})\left[\alpha \right]=\left[v_{iL},v_{iR}\right]$, where *P*_*L*_(*h*_*j*_|***v***) and *P*_*R*_(*h*_*j*_|***v***) are the conditional probabilities with respect to the lower and upper bounds of the parameters governing the FGBRBM, and *v*_*iL*_ and *v*_*iR*_ are the probabilities of the normal distribution. Consequently,

$$\begin{array}{c} \begin{array}{c} P_{L}\left(h_{j}|v\right)=P\left(h_{j}|v;\theta_{L}\right)=\sigma \left(c_{j}^{L}+W_{\cdot j}^{L}v\right),\\ P_{R}\left(h_{j}|v\right)=P\left(h_{j}|v;\theta_{R}\right)=\sigma \left(c_{i}^{R}+W_{\cdot j}^{R}v\right), \end{array} \end{array}$$ (23)

and

$$\begin{array}{c} \begin{array}{c} v_{iL}=N(b_{i}^{L}+\sigma_{i}\sum_{j=1}^{F}W_{ij}^{L}h_{j},\sigma_{i}^{2}),\\ v_{iR}=N(b_{i}^{R}+\sigma_{i}\sum_{j=1}^{F}W_{ij}^{R}h_{j},\sigma_{i}^{2}), \end{array} \end{array}$$ (24)

where $W_{ij}^{L}$ and $W_{ij}^{R}$ are the lower and upper bounds of the connection weight, respectively; $b_{i}^{L}$ and $b_{i}^{R}$ are the lower and upper bounds of visible node bias, respectively; and $c_{j}^{L}$ and $c_{j}^{R}$ are the lower and upper bounds of the hidden node bias, respectively.

The CD-1 algorithm can also be used for learning parameters of the fuzzy Gaussian-Bernoulli RBM model. In the process of training, the input data of the whole training set should first be normalized so that each dimension of the input data in the FGBRBM model obeys the normal distribution, i.e., the mean value is 0 and the variance is 1. When the formula $P(v_{i}=1|h,\bar{\theta })$ is calculated, *σ* = 1. In the process of reconstructing the nodes of the visual layer, this paper does not adopt binary data instead of the probabilities of the normal distribution. The above description is summarized as Algorithm 2.

**Algorithm 2** The learning algorithm of FGBRBM

**Input**: ***x***^(0)^ is a sample of the training dataset;

  *ε* is the learning rate;

  ***W***^*L*^ and ***W***^*R*^ are the lower and upper bounds of connection weight matrices of the visible and hidden layers;

  ***b***^*L*^ and ***b***^*R*^ are the lower and upper bounds of bias vectors of the visible nodes;

  ***c***^*L*^ and ***c***^*R*^ are the lower and upper bounds of bias vectors of the hidden nodes.

**Output**: The latest parameters of FGBRBM: ***W***^*L*^, ***W***^*R*^, ***b***^*L*^, ***b***^*R*^, ***c***^*L*^, ***c***^*R*^.

 BEGIN

 1. For all hidden nodes j do

  obtain $P_{L}\left(h_{j}^{L(0)}=1|v^{\left(0\right)}\right)$ and $P_{R}\left(h_{j}^{R(0)}=1|v^{\left(0\right)}\right)$ by means of formula (23);

  sample $h_{j}^{L(0)}\in \left\{0,1\right\}$ from $P_{L}\left(h_{j}^{L(0)}|v^{\left(0\right)}\right)$;

  sample $h_{j}^{R(0)}\in \left\{0,1\right\}$ from $P_{R}\left(h_{j}^{R(0)}|v^{\left(0\right)}\right)$;

  end for

 2. For all visible nodes i do

  obtain *v*_*iL*_ and *v*_*iR*_ by means of formula (24);

  $v_{i}^{L(1)}=N(b_{i}^{L}+\sum_{j=1}^{F}W_{ij}^{L}h_{j}^{L(0)},1)$;

  $v_{i}^{R(1)}=N(b_{i}^{R}+\sum_{j=1}^{F}W_{ij}^{R}h_{j}^{R(0)},1)$;

  end for

 3. For all hidden nodes j do

  obtain $P_{L}\left(h_{j}^{L(1)}=1|v^{L(1)}\right)$ and $P_{R}\left(h_{j}^{R(1)}=1|v^{R(1)}\right)$ by means of formula (23);

  sample $h_{j}^{L(1)}\in \left\{0,1\right\}$ from $P_{L}\left(h_{j}^{L(1)}|v^{L(1)}\right)$;

  sample $h_{j}^{R(1)}\in \left\{0,1\right\}$ from $P_{R}\left(h_{j}^{R(1)}|v^{R(1)}\right)$;

  end for

 4. The parameters can be updated according to the following rules:

  ***W***^*L*^ = ***W***^*L*^ + *ε*(***v***^(0)^ ⋅ *P*_*L*_(***h***^*L*(0)^ = 1|***v***^(0)^) − ***v***^*L*(1)^ ⋅ *P*_*L*_(***h***^*L*(1)^ = 1|***v***^*L*(1)^));

  ***b***^*L*^ = ***b***^*L*^ + *ε*(***v***^(0)^ − ***v***^*L*(1)^)

  ***c***^*L*^ = ***c***^*L*^ + *ε*(*P*_*L*_(***h***^*L*(0)^ = 1|***v***^(0)^ − *P*_*L*_(***h***^*L*(1)^ = 1|***v***^*L*(1)^));

  ***W***^*R*^ = ***W***^*R*^ + *ε*(***v***^(0)^ ⋅ *P*_*R*_(***h***^*R*(0)^ = 1|***v***^(0)^) − ***v***^*R*(1)^ ⋅ *P*_*R*_(***h***^*R*(1)^ = 1|***v***^*R*(1)^));

  ***b***^*R*^ = ***b***^*R*^ + *ε*(***v***^(0)^ − ***v***^*R*(1)^)

  ***c***^*R*^ = ***c***^*R*^ + *ε*(*P*_*R*_(***h***^*R*(0)^ = 1|***v***^(0)^ − *P*_*R*_(***h***^*R*(1)^ = 1|***v***^*R*(1)^));

 5. return ***W***^*L*^, ***W***^*R*^, ***b***^*L*^, ***b***^*R*^, ***c***^*L*^, ***c***^*R*^.

 END

*C*. *FLR and Its Learning Algorithm Used for Modeling the Click Through Rate*

The logistic regression (LR) model [24] is a classic model in the field of advertising click-rate prediction. The LR model is described in S4 Fig. The activation function of the node in the output layer is a sigmoid function [25]. The output value of the node (value of the activation function) *h*_*θ*_(***x***′) is the probability that a user will click on the advertisement; it can also be described as *P*(*y* = 1|***x***′; ***θ***′). When a sample ($x_{d}^{\prime },y_{d}$) in the training set $D=\left(x_{1}^{\prime },y_{1}\right),\left(x_{2}^{\prime },y_{2}\right),...,\left(x_{N}^{\prime },y_{N}\right)$ is given, the probability value of advertising click rate prediction can be obtained in terms of logistic regression and can be described by formula (25)

$$\begin{array}{c} p\left(click|a,u,c\right)=p\left(y_{d}=1|x_{d}^{\prime };\theta^{\prime }\right)=\frac{1}{1+e^{-\theta^{\prime T}x_{d}^{\prime }}}. \end{array}$$ (25)

Correspondingly, $p\left(y=0|x^{\prime };\theta^{\prime }\right)=1-\frac{1}{1+e^{-\theta^{\prime T}x_{d}^{\prime }}}$.

In this formula, ***θ***′^*T*^ denotes the vector of parameters of the standardized logistic regression model, ***a*** refers to the features of the advertisement, ***u*** denotes the features of users, and ***c*** represents the features of the context environment. These three features compose a vector, which can be denoted as $x_{d}^{{}^{\prime }}$. This vector is fed into the logistic regression model for CTR prediction.

For a single sample ($x_{d}^{{}^{\prime }},y_{d}$), the probability of correctly predicting the advertising click-through rate can be described as $P\left(y_{d}|x_{d}^{\prime };\theta^{\prime }\right)=h_{\theta^{\prime }}{\left(x_{d}^{\prime }\right)}^{y_{d}}{\left(1-h_{\theta^{\prime }}\left(x_{d}^{\prime }\right)\right)}^{\left(1-y_{d}\right)}$. This paper selects the likelihood function as the objective function:

$$\begin{array}{c} \begin{array}{cc} J(x_{d}^{\prime };\theta^{\prime }) & =-\frac{1}{N}log\left(P\left(Y_{D}|X_{D}^{\prime };\theta^{\prime }\right)\right)\\ & =-\frac{1}{N}log(\prod_{d=1}^{N}\left(h_{\theta^{\prime }}{\left(x_{d}^{\prime }\right)}^{y_{d}}{\left(1-h_{\theta^{\prime }}\left(x_{d}^{\prime }\right)\right)}^{1-y_{d}}\right))\\ & =-\frac{1}{N}\sum_{d=1}^{N}\left(y_{d}logh_{\theta^{\prime }}\left(x_{d}^{\prime }\right)+\left(1-y_{d}\right)log\left(1-h_{\theta^{\prime }}\left(x_{d}^{\prime }\right)\right)\right). \end{array} \end{array}$$ (26)

In formula (26), the click label *y*_*d*_ denotes the expected output and $h_{\theta^{\prime }}\left(x_{d}^{\prime }\right)$ is the real output value of the neuron node in the output layer (output of the activation function); $h_{\theta^{\prime }}\left(x_{d}^{\prime }\right)$ can be described as $h_{\theta^{\prime }}\left(x_{d}^{\prime }\right)=g\left(z\right)$, where $z=\sum_{i}\left(w_{i}x_{i}+b\right)=\theta^{\prime T}x_{d}^{\prime }$ and *g*(*x*) denotes the *sigmoid* function.

As for the fuzzy logistic regression model, the objective function can be described as

$$\begin{array}{c} \begin{array}{cc} \bar{J}\left(x_{d}^{\prime };\bar{\theta^{\prime }}\right) & =-\frac{1}{N}log\left(\bar{P}\left(Y_{D}|X_{D}^{\prime };\bar{\theta^{\prime }}\right)\right)\\ & =-\frac{1}{N}log(\prod_{d=1}^{N}\left({\bar{h}}_{\bar{\theta^{\prime }}}{\left(x_{d}^{\prime }\right)}^{y_{d}}{\left(1-{\bar{h}}_{\bar{\theta^{\prime }}}\left(x_{d}^{\prime }\right)\right)}^{1-y_{d}}\right))\\ & =-\frac{1}{N}\sum_{d=1}^{N}\left(y_{d}log{\bar{h}}_{\bar{\theta^{\prime }}}\left(x_{d}^{\prime }\right)+\left(1-y_{d}\right)log\left(1-{\bar{h}}_{\bar{\theta^{\prime }}}\left(x_{d}^{\prime }\right)\right)\right), \end{array} \end{array}$$ (27)

where ${\bar{h}}_{\bar{\theta^{\prime }}}\left(x_{d}^{\prime }\right)=\bar{g}\left(\bar{z}\right)$, $\bar{z}=\sum_{i}\left(\bar{w_{i}}x_{i}+\bar{b}\right)={\bar{\theta^{\prime }}}^{T}x_{d}^{\prime }$ and $g\left(x\right)=\frac{1}{1+e^{-x}}$.

According to formulas (5) and (7), the gradient of the objective function of FLR with respect to fuzzy parameter $\bar{\theta^{\prime }}$ can be expressed as

$$\begin{array}{c} \begin{array}{cc} \frac{\partial \bar{J}\left(x_{d}^{\prime };\bar{\theta^{\prime }}\right)}{\partial {\bar{\theta^{\prime }}}_{L}} & =\frac{\partial J_{c}\left(x_{d}^{\prime };\bar{\theta^{\prime }}\right)}{\partial {\bar{\theta^{\prime }}}_{L}}\\ & =-\frac{1}{N}\sum_{d=1}^{N}\left(y_{d}\frac{1}{h_{\theta_{L}^{\prime }}\left(x_{d}^{\prime }\right)}\frac{\partial h_{\theta_{L}^{\prime }}\left(x_{d}^{\prime }\right)}{\partial \theta_{L}^{\prime }}-\left(1-y_{d}\right)\frac{1}{1-h_{\theta_{L}^{\prime }}\left(x_{d}^{\prime }\right)}\frac{\partial h_{\theta_{L}^{\prime }}\left(x_{d}^{\prime }\right)}{\partial \theta_{L}^{\prime }}\right)\\ & =\frac{1}{N}\sum_{d=1}^{N}(h_{\theta_{L}^{\prime }}\left(x_{d}^{\prime }\right)-y_{d})x_{d}^{\prime }\\ & =C\sum_{d=1}^{N}(h_{\theta_{L}^{\prime }}\left(x_{d}^{\prime }\right)-y_{d})x_{d}^{\prime }, \end{array} \end{array}$$ (28)

$$\begin{array}{c} \begin{array}{cc} \frac{\partial \bar{J}\left(x_{d}^{\prime };\bar{\theta^{\prime }}\right)}{\partial {\bar{\theta^{\prime }}}_{R}} & =\frac{\partial J_{c}\left(x_{d}^{\prime };\bar{\theta^{\prime }}\right)}{\partial {\bar{\theta^{\prime }}}_{R}}\\ & =-\frac{1}{N}\sum_{d=1}^{N}\left(y_{d}\frac{1}{h_{\theta_{R}^{\prime }}\left(x_{d}^{\prime }\right)}\frac{\partial h_{\theta_{R}^{\prime }}\left(x_{d}^{\prime }\right)}{\partial \theta_{R}^{\prime }}-\left(1-y_{d}\right)\frac{1}{1-h_{\theta_{R}^{\prime }}\left(x_{d}^{\prime }\right)}\frac{\partial h_{\theta_{R}^{\prime }}\left(x_{d}^{\prime }\right)}{\partial \theta_{R}^{\prime }}\right)\\ & =\frac{1}{N}\sum_{d=1}^{N}(h_{\theta_{R}^{\prime }}\left(x_{d}^{\prime }\right)-y_{d})x_{d}^{\prime }\\ & =C\sum_{d=1}^{N}(h_{\theta_{R}^{\prime }}\left(x_{d}^{\prime }\right)-y_{d})x_{d}^{\prime }. \end{array} \end{array}$$ (29)

The above description is summarized as Algorithm 3.

**Algorithm 3** The learning algorithm of FLR

**Input**: $\left(x_{d}^{\prime },y_{d}\right)$ is a sample of training sets;

  *ε* is the learning rate;

  $\theta_{L}^{\prime }=\left\{W^{L},c^{L}\right\}$ and $\theta_{R}^{\prime }=\left\{W^{R},c^{R}\right\}$, where ***W***^*L*^ and ***W***^*R*^ are the lower and upper bounds of connection weight matrices of FLR, and ***c***^*L*^ and ***c***^*R*^ are the lower and upper bounds of bias vectors of the output node of FLR;

**Output**: The latest parameters of FLR: $\theta_{L}^{\prime }=\left\{W^{L},c^{L}\right\}$, $\theta_{R}^{\prime }=\left\{W^{R},c^{R}\right\}$.

 BEGIN

 1. obtain $\frac{\partial J_{c}\left(\bar{\theta^{\prime }}\right)}{\partial \theta_{L}^{\prime }}$ by means of formula (28) based on the input data $x_{d}^{\prime }$;

 2. obtain $\frac{\partial J_{c}\left(\bar{\theta^{\prime }}\right)}{\partial \theta_{R}^{\prime }}$ by means of formula (29) based on the input data $x_{d}^{\prime }$;

 3. The parameters can be updated according to the following rules:

  $\theta_{L}^{\prime }=\theta_{L}^{\prime }+\varepsilon \frac{\partial J_{c}\left(\bar{\theta^{\prime }}\right)}{\partial \theta_{L}^{\prime }}$;

  $\theta_{R}^{\prime }=\theta_{R}^{\prime }+\varepsilon \frac{\partial J_{c}\left(\bar{\theta^{\prime }}\right)}{\partial \theta_{R}^{\prime }}$;

 4. return $\theta_{L}^{\prime },\theta_{R}^{\prime }$.

 END

#### Advertising CTR prediction model FDNN

*A*. *Construction of Advertising CTR Prediction Model FDNN*

This section describes the proposed fuzzy deep neural network (FDNN) model for advertising CTR prediction and its learning algorithm in detail. The architecture of the method and its process of predicting CTR are illustrated in S5 Fig.

First, the fuzzy deep belief network (FDBN) constituted by FRBM and GBRBM is used to automatically learn the discriminative features of raw input data from an advertising dataset, and these features are able to capture internal explanatory information that is hidden in the raw data, amplify the important information for discrimination and weaken irrelevant information [26].

Next, FLR is adopted to model the CTR prediction and map the predicted CTR value to the range between 0 and 1.

The raw input data of advertising CTR prediction are the fields that include the corresponding advertisement features, users’ features, and context features, which are extracted from the advertisement click log. These fields contain not only numerical data but also multi-category data; therefore, the raw input data of advertising CTR prediction are composed of vectors with different data types.

Because FRBM only models input with binary variance (0, 1), this paper adopts fuzzy Gaussian-Bernoulli RBM (FGBRBM) to address the input data of advertising CTR prediction.

Then, FRBM can be used to construct FDBN through layer-by-layer stacking; the detailed steps can be found in [22].

In real applications, advertising click rate prediction refers to binary classification or regression; therefore, this paper uses fuzzy logistic regression to model user behaviors in clicking advertisements. After the input data vector ***x*** = (***a***, ***u***, ***c***) = (*x*_1_, *x*_2_, …, *x*_*n*_) is extracted by FDBN, the vector $x^{{}^{\prime }}$, which is composed of the binary values, in the last hidden layer of FDBN is fed into fuzzy the logistic regression model for CTR prediction.

The advantage of the proposed method is that fuzzy set techniques are introduced, and the uncertainties in the relationships between nodes located in adjacent layers is taken into consideration. Nodes in adjacent layers of FDNN often interact in uncertain ways. Because the parameters that represent the relationships between nodes in adjacent layers are fuzzy numbers and the learning process of fuzzy parameters is extended to a relatively wider space, this advantage will be reflected in the fitness of the joint probability distribution. Combined with the merits of deep learning, the proposed method demonstrates superior performance in coping with data with noises.

*B*. *Construction and Training of the Advertising CTR Prediction Model FDNN*

The process of constructing FDNN is illustrated in S6 Fig, and the detailed steps of constructing the FDNN are as follows:

Step 1: The training of FRBM layer by layer

First, the vectors that are constituted by related data of textual advertising click logs are regarded as the input of FGBRBM, and the CD1 algorithm is used to train FGBRBM; in this way, the parameter ***θ***_1_ of FGBRBM is obtained. Based on the input vectors and the parameter ***θ***_1_, the hidden nodes ***h***_1_ of FGBRBM are obtained.

Second, the parameter ***θ***_2_ of the current FRBM can be obtained when ***h***_1_ is regarded as the input data for the CD1 algorithm. Based on ***h***_1_ and ***θ***_2_, the hidden nodes ***h***_2_ of the current FRBM can be obtained.

Third, the previous process is repeated until reaching the fixed layer.

Step 2: Constructing FDBN by means of FRBMs

According to the training procedure of FRBM in step 1, the weight values of FRBMs are connected from bottom to top to construct FDBN. In this FDBN, the connection between the top two layers is undirected and the other connection is directed from top to bottom [22].

Step 3: Constructing the FDNN that is used for advertising CTR prediction by means of FDBN

After an FLR model is added onto the top of the FDBN, the probability that the user clicks this advertisement is equal to the output value of the nodes of the input layer. At this point, the network becomes the initially trained fuzzy deep neural network (FDNN).

Step 4: Fine-tuning the parameters of FDNN

After FDNN is initially trained layer by layer, a fine-tuning process needs to be conducted. In contrast to the unsupervised learning approach in the initial training, the fine-tuning is implemented using supervised learning. Based on the weight values of the network that were obtained from the initial training, error backpropagation [27, 29] and stochastic gradient descent are used to update the weight values and bias of the neural network. After fine-tuning, the process of training FDNN is complete. The output of FDNN is in the form of a probability: $p\left(click|a,u,c\right)=p\left(y_{d}=1|x_{d}^{\prime };\theta^{\prime }\right)$.

The process of fine-tuning the parameters of FDNN contains two phases.

1) Forward calculation of the input signal:

When labeled data (***x***_*d*_, *y*_*d*_) of click logs are given, that is, the combined feature vector ***x*** = (***a***, ***u***, ***c***) = (*x*_1_, *x*_2_, …, *x*_*n*_) is given, the jth node in the lth layer (*l* = 2, ‥, *L*_*n*_) of the FDNN deep neural network can be defined as follows:

*L*_*n*_ denotes the total number of layers in the FDNN network;

*s*_*l*_ denotes the number of nodes in the lth layer of the FDNN network;

$\bar{w_{ji}^{l}}$ denotes the connection weight between the *j*th node in the *l*th layer and the *i*th node in the *l* − 1th layer; $\bar{b_{j}^{l}}$ denotes the bias of the *j*th node in the *l*th layer; $\bar{net_{j}^{l}}=\bar{b_{j}^{l}}+\sum_{i=1}^{s_{l-1}}\bar{w_{ji}^{l}}\bar{o_{i}^{l-1}}$ denotes the weighted-sum input of the *j*th node in the *l*th layer;

$\bar{o_{j}^{l}}$ denotes the output value of the activation function of the *j*th node in the *l*th layer;

The selected activation function is sigmoid function.

In the forward calculation, the input signal is transmitted from the input layer to the output layer. The input value of the *j*th node in the second layer of FDNN (*l* = 2) can be described as $\bar{net_{j}^{2}}=\bar{b_{j}^{2}}+\sum_{i=1}^{s_{1}}\bar{w_{ji}^{2}}x_{i}$. When the input of node j is transferred by the activation function, its output value can be described as $\bar{o_{j}^{2}}=g\left(\bar{net_{j}^{2}}\right)$. Therefore, the input value of the *j*th node in the *l*th layer of FDNN (*l* = 3, …, *L*_*n*_) can be described as $\bar{net_{j}^{l}}=\bar{b_{j}^{l}}+\sum_{i=1}^{s_{l-1}}\bar{w_{ji}^{l}}o_{i}$ and its corresponding output value can be described as $\bar{o_{j}^{l}}=g\left(\bar{net_{j}^{l}}\right)$.

In particular, the output value of the jth node in the th layer of FDNN can be described as

$$\begin{array}{c} {\bar{h}}_{\bar{\theta }}\left(x_{d}\right)=O_{j}=O_{1}=\bar{O_{1}^{L_{n}}}. \end{array}$$ (30)

2) Backpropagation of the error signal:

This paper regards the cross-entropy as the objective function [25]. It can be described as formula (31). The local gradient will be refined using backpropagation. The error signal between the output value and labeled signal will be transmitted from the output layer to the input layer in the process.

$$\begin{array}{c} \begin{array}{c} \begin{array}{cc} \bar{J}\left(x_{d};\bar{\theta }\right) & =y_{d}log{\bar{h}}_{\bar{\theta }}\left(x_{d}\right)+\left(1-y_{d}\right)log\left(1-{\bar{h}}_{\bar{\theta }}\left(x_{d}\right)\right)\\ & =y_{d}logg\left(\bar{net_{j}^{L_{n}}}\right)+\left(1-y_{d}\right)log\left(1-g\left(\bar{net_{j}^{L_{n}}}\right)\right), \end{array} \end{array} \end{array}$$ (31)

In this formula, *y*_*d*_ is the label value of the sample, which can be represented as *y*_*d*_ ∈ [0, 1].

The gradient descent method will be adopted to learn the model. This paper will first define the residual error $\bar{\delta_{j}^{l}}$ [28] of the *j*th node in the *l*th layerlayer as the partialand of objective function $\bar{J}$ with respect to the weighted sum $\bar{net_{j}^{l}}$ of node *j*. The residual error $\bar{\delta_{j}^{L_{n}}}$ of the *j* th neuron node of the *L*_*n*_ th layer can be calculated by formula (32)

$$\begin{array}{c} \begin{array}{cc} \bar{\delta_{j}^{L_{n}}} & =\frac{\partial \bar{J}}{\partial \bar{net_{j}^{L_{n}}}}=y_{d}\frac{1}{g(\bar{net_{j}^{L_{n}}})}g^{\prime }\left(\bar{net_{j}^{L_{n}}}\right)+\left(1-y_{d}\right)\frac{-1}{1-g(\bar{net_{j}^{L_{n}}})}g^{\prime }\left(\bar{net_{j}^{L_{n}}}\right)\\ & =y_{d}-g\left(\bar{net_{j}^{L_{n}}}\right)\\ & =y_{d}\bar{O_{1}^{L_{n}}}. \end{array} \end{array}$$ (32)

The residual error $\delta_{j}^{l}$ of the *j*th neuron node of the *l*th layer (*l* = 2, …, *L*_*n*_ − 1) can be calculated by formula (33) according to the chain rule [29].

$$\begin{array}{c} \begin{array}{c} \begin{array}{cc} \bar{\delta_{j}^{l}} & =\frac{\partial \bar{J}}{\partial \bar{net_{j}^{l}}}=\frac{\partial \bar{J}}{\partial \bar{o_{j}^{l}}}\cdot \frac{\partial \bar{o_{j}^{l}}}{\partial \bar{net_{j}^{l}}}\\ & =\sum_{i=1}^{s_{l+1}}(\frac{\partial \bar{J}}{\partial \bar{net_{i}^{l+1}}}\frac{\partial \bar{net_{i}^{l+1}}}{\partial \bar{o_{j}^{l}}})\cdot \frac{\partial \bar{o_{j}^{l}}}{\partial \bar{net_{j}^{l}}}\\ & =(\sum_{i=1}^{s_{l+1}}\bar{\delta_{i}^{l+1}}\cdot \bar{w_{ji}^{l+1}})\frac{\partial g(\bar{net_{j}^{l}})}{\partial \bar{net_{j}^{l}}}\\ & =(\sum_{i=1}^{s_{l+1}}\bar{\delta_{i}^{l+1}}\cdot \bar{w_{ji}^{l+1}})g\left(\bar{net_{j}^{l}}\right)\left(1-g\left(\bar{net_{j}^{l}}\right)\right)\\ & =(\sum_{i=1}^{s_{l+1}}\bar{\delta_{i}^{l+1}}\cdot \bar{w_{ji}^{l+1}})\bar{o_{j}^{l}}\left(1-\bar{o_{j}^{l}}\right), \end{array} \end{array} \end{array}$$ (33)

where $\bar{w_{ji}^{l+1}}$ denotes the connection weight between the *j*th node of the *l*th layer and the *i*th node of *l* + 1th layer.

According to formulas (2) and (10), the gradient of cost function $\bar{J}\left(\bar{W},\bar{b}\right)$ with respect to parameters $\bar{w_{ji}^{l}}$ and $\bar{b_{j}^{l}}$ can be calculated by formulas (34), (35), (36) and (37).

$$\begin{array}{c} \begin{array}{c} \frac{\partial \bar{J}}{\partial {w_{ji}^{l}}_{L}}=\frac{\partial \bar{J}}{\partial \bar{net_{j}^{l}}}\cdot \frac{\partial \bar{net_{j}^{l}}}{\partial {w_{ji}^{l}}_{L}}={\bar{\delta_{j}^{l}}}_{L}\cdot {\bar{o_{i}^{l-1}}}_{L}, \end{array} \end{array}$$ (34)

$$\begin{array}{c} \begin{array}{c} \frac{\partial \bar{J}}{\partial {b_{j}^{l}}_{L}}={\bar{\delta_{j}^{l}}}_{L}, \end{array} \end{array}$$ (35)

$$\begin{array}{c} \begin{array}{c} \frac{\partial \bar{J}}{\partial {w_{ji}^{l}}_{R}}=\frac{\partial \bar{J}}{\partial \bar{net_{j}^{l}}}\cdot \frac{\partial \bar{net_{j}^{l}}}{\partial {w_{ji}^{l}}_{R}}={\bar{\delta_{j}^{l}}}_{R}\cdot {\bar{o_{i}^{l-1}}}_{R}, \end{array} \end{array}$$ (36)

$$\begin{array}{c} \begin{array}{c} \frac{\partial \bar{J}}{\partial {b_{j}^{l}}_{R}}={\bar{\delta_{j}^{l}}}_{R}. \end{array} \end{array}$$ (37)

The above process is described in Algorithm 4.

**Algorithm 4** The algorithm for fine-tuning the parameters of FDNN using BP

**Input**: (***x***_*d*_, *y*_*d*_) is the labeled training data of textual advertising click logs;

  *ε* is the learning rate;

  ${w_{ji}^{l}}_{L}$ is the lower bound of the connection weight between the *j*th node in the *l*th layer and the *i*th node in the (*l* − 1)th layer of FDNN;

  ${b_{j}^{l}}_{L}$ is the lower bound of the bias of the *j*th node in the *l*th layer of FDNN;

  ${w_{ji}^{l}}_{R}$ is the upper bound of the connection weight between the *j*th node in the *l*th layer and the *i*th node in the (*l* − 1)th layer of FDNN;

  ${b_{j}^{l}}_{R}$ is the upper bound of the bias of the *j*th node in the *l*th layer of FDNN;

**Output**: The latest parameters: ${w_{ji}^{l}}_{L}$, ${w_{ji}^{l}}_{R}$, ${b_{j}^{l}}_{L}$, ${b_{j}^{l}}_{R}$

 BEGIN

 1. In the forward-propagation calculation, when the input data vector ***x***_*d*_ is fed into the input layer, the output value of FDNN can be obtained using formula (30);

 2. In the calculation of error backpropagation,

  obtain $\frac{\partial \bar{J}}{\partial {w_{ji}^{l}}_{L}}$ by means of formula (34);

  obtain $\frac{\partial \bar{J}}{\partial {b_{j}^{l}}_{L}}$ by means of formula (35);

  obtain $\frac{\partial \bar{J}}{\partial {w_{ji}^{l}}_{R}}$ by means of formula (36);

  obtain $\frac{\partial \bar{J}}{\partial {b_{j}^{l}}_{R}}$ by means of formula (37);

 3. The parameters can be updated according to the following rules:

  ${w_{ji}^{l}}_{L}={w_{ji}^{l}}_{L}+\varepsilon \frac{\partial \bar{J}}{\partial {w_{ji}^{l}}_{L}}$;

  ${b_{j}^{l}}_{L}={b_{j}^{l}}_{L}+\varepsilon \frac{\partial \bar{J}}{\partial {b_{j}^{l}}_{L}}$;

  ${w_{ji}^{l}}_{R}={w_{ji}^{l}}_{R}+\varepsilon \frac{\partial \bar{J}}{\partial {w_{ji}^{l}}_{R}}$;

  ${b_{j}^{l}}_{R}={b_{j}^{l}}_{R}+\varepsilon \frac{\partial \bar{J}}{\partial {b_{j}^{l}}_{R}}$;

 4. return ${w_{ji}^{l}}_{L}$, ${b_{j}^{l}}_{L}$, ${w_{ji}^{l}}_{R}$, ${b_{j}^{l}}_{R}$.

 END

### Experiment datasets and environment

#### Description of dataset

This paper uses the Criteo Display Advertising Challenge dataset, which is provided by the Criteo company on the famous machine learning website Kaggle for advertising CTR competition [30]. Every advertisement log record contains the set of features of the displayed advertisement, which is composed of 13 numerical features and 26 categorical features. The detailed formats of these features are given in S1 Table. The class Label is used to indicate whether this advertisement is clicked: if the advertisement is clicked, the value of Label is 1; otherwise, the value of Label is 0. Categorical features are desensitized using Harsh mapping; therefore, real meaning can not be obtained from them.

To adequately evaluate the performance of this model and prevent the interference of many factors such as data timeliness, we randomly disrupt the order of the raw samples in the experiment. Meanwhile, to avoid the over-fitting, the 5-fold cross validation method [31] is adopted in the training process of all experiments.

#### Experiment environment

Six high-performance workstations are used for the experiments. The hardware and software configurations of the experimental environment are shown as S2 Table.

## Results and analysis

### Baseline models

This paper selects the logistic regression (LR), Factorization Machines (FMs) [32], GBDT+FM [8], and DBNLR [11] models as the baseline models for performance comparison.

### Performance evaluation metric of models

#### AUC

This paper uses the area under the receiver operating characteristic curve (AUC) [33, 34] as the main performance evaluation metric for advertising CTR prediction. The confusion matrix is shown as S3 Table [35, 36]. Formulas *TPR* = *TP*/(*TP* + *FN*) and *FPR* = *FP*/(*FP* + *TN*) are used to calculate the corresponding TPR and FPR values of the confusion matrix to obtain a co-ordinate pair (dot pair). The dot pairs make up the receiver operating characteristic (ROC) curve [37]. The AUC is the total area under the ROC. The larger the AUC is, the more accurate the advertising CTR prediction is, and the better the effect achieved by the advertising placement is.

#### Log-loss

This paper selects the logarithmic loss function ‘log-loss’ [38, 39] as another auxiliary performance evaluation metric for advertising CTR prediction.

$$\begin{array}{c} \begin{array}{c} logloss=-\frac{1}{L}\sum_{i=1}^{L}y_{i}log{\bar{y}}_{i}+\left(1-y_{i}\right)log\left(1-{\bar{y}}_{i}\right), \end{array} \end{array}$$ (38)

where *L* is the number of samples, *y*_*i*_ is the true label (0 or 1), and ${\bar{y}}_{i}$ is the predicted probability. The value of log-loss reflects the similarity between the CTR predicted by model and real CTR. The smaller the value of log-loss is, the more accurate the advertising CTR prediction is.

### Experiments design and results analysis

#### Configuration optimization and performance analysis of FDNN and DBNLR

*A*. *Feature Pre-processing*

FDNN is similar with DBNLR in the architecture. Their difference lies in the components of model. Therefore, this paper selects the DBNLR as a baseline model.

In the experiment on the FDNN and DBNLR models, the numerical features of the raw dataset are directly adopted, and these categorical features are expanded to binary vectors using one-hot representation.

Although the method of one-hot representation is straightforward and effective, it makes the feature vectors sparse and high-dimensional. In the deep learning models, when the dimensionality of the features is large, the scale of the parameters of the model becomes large, which leads to a sharp increase in training time. Moreover, the hardware will be a bottleneck in the training process. Therefore, this paper adopts a feature hashing strategy (signed hashing trick) [40] to reduce the dimensionality of these sparse binary feature vectors. Finally, the joint vector that is composed of numerical features and categorical features and has been processed by the feature hashing strategy is regarded as the input of the two deep architecture models.

Input vectors can be obtained when the data of the Display Advertising Challenge dataset are preprocessed using the feature preprocessing methods described above. In this experiment, after preprocessing, the dimensionality of the input vector is 1746088. Therefore, the number of nodes in the visible layer (input layer) is 1746088.

*B*. *The Parameter Settings for Training FDNN and DBNLR*

These two models are developed based on the Theano framework [41] by means of Python. In all experiments, the maximum number of learning epochs in the training process is set to a sufficiently large value (800) and the Early-Stopping strategy is adopted to automatically cut epochs for obtaining the best validation score. Furthermore, this strategy can also avoid over-fitting.

The steps of the Early-Stopping strategy are as follows:

1. Split the dataset into a training set and a validation set;

2. When the training of every epoch has been finished, calculate the validation error (loss) in the validation dataset;

3. When the validation error of the validation dataset no longer decreases, record the number of epochs and the current validation error value. If the validation error of the validation dataset does not increase after continuation, the training process is terminated, the best validation score is obtained, and optimization is complete. Meanwhile, the optimal parameter estimation values are obtained.

In the initial training process of FDBN and DBNLR, the gradient descent method with minibatch is used for training on these samples and each minibatch includes 2000 training samples. For FGBRBM (or GBRBM), the learning rate is 0.01, while for FRBM (or RBM) with the binary variable, the learning rate is 0.1. The decay factor of the weight value is 0.0002.

In the fine-tuning process of FDNN and DBNLR, the gradient descent method with minibatch is used for training on these samples and each minibatch includes 4000 training samples. The decay factor of the weight value is 0 and the learning rate is set as 0.005.

Dropout represents the probability that a node is retained in the neural network [42, 43]. A reasonable sparse dropout strategy can strengthen the robustness of a neural network. Dropout is utilized to reduce over-fitting and the complexity of these two deep learning models. The paper sets the dropout rate to 0.5, 0.6, 0.7, 0.8, and 0.9 to implement the testing experiments. Under the condition of having the same configuration structure, the dropout rates are respectively 0.5 and 0.6 when FDNN and DBNLR use their own optimal settings.

*C*. *Optimization of the number of hidden layers and hidden nodes*

Currently, there is no method for quickly setting the number of hidden layers and the number of hidden nodes in each layer. Therefore, many experiments and much experience are utilized to search for the optimal structure. According to past experience, when the number of nodes of the visible layer is large, the nodes of the hidden layer need to perform “dimensionality reduction- dimensionality addition—dimensionality reduction”. However, when the number of nodes of the visible layer is small, the nodes of the hidden layer only need to perform “dimensionality addition—dimensionality reduction”. In general, the number of hidden nodes will be increased or decreased with multiple. By comparing the performances in many experiments with different configurations (different numbers of nodes of each hidden layer and numbers of hidden layers), the configuration with relatively optimal performance can be obtained.

In S4 Table, information on different configurations in many experiments is given. In S7 Fig, the corresponding AUC values of DBNLR and FDNN with different configurations are given. In S8 Fig, the corresponding log-loss values of DBNLR and FDNN with different configurations are given.

As presented in S7 and S8 Figs, in the experiments for training FDNN, in the beginning, increasing the number of hidden layers can improve the performance of the proposed method; however, when the number of hidden layers is more than 3, the performance is degraded. This is because FDNN does not adequately capture feature interactions when the number of hidden layers is small. However, when the number of hidden layers and the number of nodes in the hidden layers exceed a certain scale, cross-features disturb and degrade the accuracy of CTR prediction. The over-fitting phenomenon is observed and the algorithm spends more time learning the parameters.

After many experiments have been completed, a configuration with relatively optimal performance is obtained: Conf11, the number of hidden layers is 3; the number of nodes of the first hidden layer is 170; the number of nodes of the second hidden layer is 1700; and the number of nodes of the third hidden layer is 17. This paper chooses this configuration as the final structure of FDNN. Corresponding AUC and log-loss on the test dataset can be seen in S9 and S10 Figs.

In the experiments for training DBNLR, relatively optimal performance is obtained when the configuration structure is as follows: Conf7, the number of hidden layers is 3; the number of nodes of the first hidden layer is 150; the number of nodes of the second hidden layer is 1500; and the number of nodes of the third hidden layer is 15. This paper chooses this configuration as the final structure of DBNLR. Corresponding AUC and log-loss on the test dataset can be seen in S9 and S10 Figs.

We observe that the curve of the AUC value of the DBNLR model and that of the FDNN model are indented. However, when they have the same configuration, the overall level of performance of DBNLR is worse than that of FDNN; this is true for the log-loss index. It is because fuzzy set techniques are introduced in the proposed FDNN model. The components of FDNN model are fuzzy RBM, fuzzy GBRBM and fuzzy LR. With the data of many records in the Display Advertising Challenge dataset which contains outliers and missing values implying many uncertainties, the uncertainties can be taken into consideration and efficiently handled by fuzzy set techniques; therefore, the performance of the FDNN model for CTR prediction is better than that of DBNLR in the experiment.

#### Configuration optimization of other baseline models

*A*. *LR Solution*

CTR prediction in a real advertising system involves a large number of samples, while logistic regression (LR) model can appropriately address a large number of features and can be trained rapidly, so the LR model is selected as a baseline model in this paper. This paper selects LIBLINEAR [44] to implement L2-regularized logistic regression, and the stochastic gradient method (SG) is adopted to optimize the parameters of LR. In the LR solution, the numerical features are used directly and the categorical features are first represented with one-hot encoding and then used to establish the feature space with MurmurHash 3. After many experiments, this paper adopts the following parameters: *η* = 0.2, *λ* = 0.

*B*. *FM Solution*

For advertising CTR prediction, Factorization Machines (FMs) combine the advantages of Support Vector Machines with factorization models, and can characterize the basic feature interactions of a large number of advertising data, therefore, this paper selects the FM model as a baseline model [45]. For advertising CTR prediction, Factorization Machines (FMs) is also adopted, which is based on feature engineering and matrix design. This paper adopts LIBFM to implement factorization machines [46, 47] and the stochastic gradient method (SG) is adopted to optimize the parameters of FM. The numerical features are used directly and the categorical features are first represented with one-hot encoding. After many experiments, this paper adopts the following parameters: *λ* = 40, *k* = 30.

*C*. *GBDT+FM Solution*

Gradient Boost Decision Tree (GBDT) is a nonlinear model that is based on the concept of collective learning boosting. GBDT has strong extracting feature ability and nonlinear fitting ability, and it can flexibly handle various types of data in the advertising dataset including continuous value and discrete value, but no over-fitting occurs. Therefore, this paper selects GBDT+FM solution as a baseline model. Based on the work of Y Juan et al., this paper implements GBDT+FM solutions for performance comparison and analysis. First, in Preprocessing-A, all numerical features are used directly and all categorical features are represented with one-hot encoding. Then, these features are fed into decision trees in GBDT to generate GBDT features. This paper selects Xgboost to implement the GBDT [48]. In Preprocessing-A, the number of decision trees in GBDT is 30 and the depth is 7. Thirty features are generated for each impression. The following process is Preprocessing-B, which is used for generating input features for FM. In Preprocessing-B, numerical features (I1-I13) with values greater than 2 are transformed by *v* ← ⌊*log*(*v*)^2^⌋; categorical features (C1-C26) that appear fewer than 10 times are transformed into a special value; and GBDT features are directly included. Therefore, each record contains 69 features: 13 numerical features, 26 categorical features and 30 GBDT features. Then, these three groups of features are hashed into 1 M dimensions using a hashing trick. Finally, the processed features are fed into the FM model for CTR prediction. The parameters of the FM model that achieve the relatively optimal results are “*λ* = 40, *k* = 100”.

#### Experimental results and analysis

According to S9 and S10 Figs, the proposed FDNN method has the best performance and LR has the worst performance. In the process of training models, it can be found that logistic regression model can be trained rapidly, but its effect is in the general level. One reason is that when LR is used to predict CTR, it requires the experts with sophisticated experience to implement feature engineering through the human labor. The other reason is that the simple structure of LR restrains its representation ability. These two disadvantages cause that LR cannot learn complex feature interactions and is insensitive to outliers and missing values. Therefore, LR has the relatively bad performance in the experiment.

According to S9 and S10 Figs, FM is superior in performance to LR. The reason is that FM extends the idea of LR because FM adds to second order fitting on the basis of first order fitting and it can automatically learn cross features of any two features from different dimensions. And cross features are expressed with embedding vector. Compared with LR, FM model has fewer manual intervene but higher efficiency. Therefore, FM has better representation ability than LR in the experiment. However, FM also cannot learn much more complex feature interactions because of its relatively simple structure.

In this experiment, GBDT+FM solution outperform the FM model. The GBDT+FM solution obtains good prediction results, which are slightly inferior to those of the proposed method. The GBDT can learn useful high-order feature interactions because it has strong nonlinear fitting ability and the robustness for hyperparameter, so it can efficiently extracts discriminative features and cross-features of advertising dataset, and shows better performance than FM model in our CTR prediction experiment.

Compared with the GBDT+FM solution, the proposed method achieves more than 0.09% improvement in terms of AUC (0.01% in terms of log-loss) on the dataset. The reasons are as follows. The first is that the quality of data exerts a great influence on effect of model. Because the dataset contains many noisy data, there are bottlenecks in the effects of GBDT+FM solution. Compared with this model, the proposed FDNN model, as a result of being endowed with fuzzy technology, can capture more-complex high-order feature interactions of the advertising dataset, which contains many outliers and missing values. The second is that GBDT+FM solution, belonging to a kind of stacking combined model without backproportion, is not always better than the proposed model with backproportion in effect improvement for CTR prediction from the theoretical perspective. Therefore, it demonstrates relatively good performance in terms of both data representation capability and prediction accuracy. In a real production environment for an advertising system, a small improvement in advertising CTR is likely to result in a significant increase in financial income for the internet company and an improved user experience.

## Discussion

This paper proposes a fuzzy deep neural network (FDNN) to address the problem of advertising CTR prediction. The network’s performance is compared with those of several baseline models in real advertisement datasets. Due to the fuzziness that is introduced into the proposed model, it can learn the most useful high-order feature interactions and demonstrates good performance in terms of both data representation capability and robustness in advertisement datasets with noise.

Big data bring new perspectives to these fields and challenges in processing and discovering valuable information. The traditional artificial intelligence techniques can no longer effectively extract and organize the discriminative information from raw advertisement data directly [49]. This paper provides a basis for the further application of fuzzy deep neural networks in advertising CTR prediction. Future work will focus on exploring additional unsupervised feature learning and deep architecture models to improve the CTR prediction for internet advertising.

In addition, in big data era, a real production environment for advertising CTR prediction requires that the advertising system return the prediction results in milliseconds or even microseconds. Determining how to implement low-lag and high-efficiency impromptu Ad-Hoc analysis to predict and return results based on a big dataset is a great challenge for big data processing systems. Big data systems with stream computing, such as Spark Streaming, Storm, and Flink, can implement analysis and query in real time. However, because of the limited memory capacity and loss of raw historical data in system, Ad-Hoc analysis and query for a big dataset cannot be implemented. Therefore, exploring CTR prediction solutions based on streaming big data processing technology is another future direction of the work of this paper.

## Supporting information

S1 Fig Triangular fuzzy number. (ZIP)

S2 Fig Structure of fuzzy restricted boltzmann machine. (ZIP)

S3 Fig Structure of fuzzy gaussian-bernoulli restricted boltzmann machine. (ZIP)

S4 Fig Modeling click-through rate prediction by means of LR. (ZIP)

S5 Fig Architecture of the FDNN model and its process of predicting CTR. (ZIP)

S6 Fig The process of constructing FDNN. (ZIP)

S7 Fig AUC values of FDNN and DBNLR with different numbers of layers and nodes. (ZIP)

S8 Fig Log-loss values of FDNN and DBNLR with different numbers of layers and nodes. (ZIP)

S9 Fig Comparison of AUC among all models on the test dataset. (ZIP)

S10 Fig Comparison of log-loss among all models on the test dataset. (ZIP)

S1 Table Data format of dataset. (ZIP)

S2 Table Experimental environment. (ZIP)

S3 Table Confusion matrix. (ZIP)

S4 Table Configure information of FDNN and DBNLR with different numbers of layers and nodes. (ZIP)
