# Supplementary figures and images for: An improved advertising CTR prediction approach based on the fuzzy deep neural network
Source: PLoS One. 2018 May 4;13(5):e0190831. doi: 10.1371/journal.pone.0190831 (PMC5935396; doi:10.1371/journal.pone.0190831)

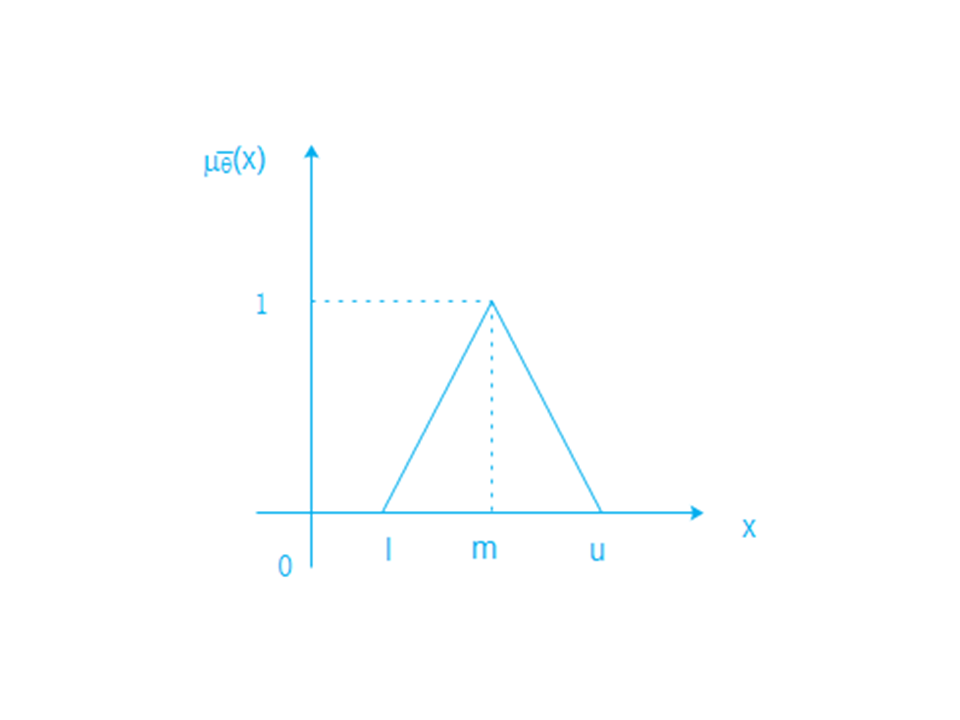

Supplement: S1 Fig — (ZIP) [file pone.0190831.s001.zip › S1 Fig/S1_Fig.tif]

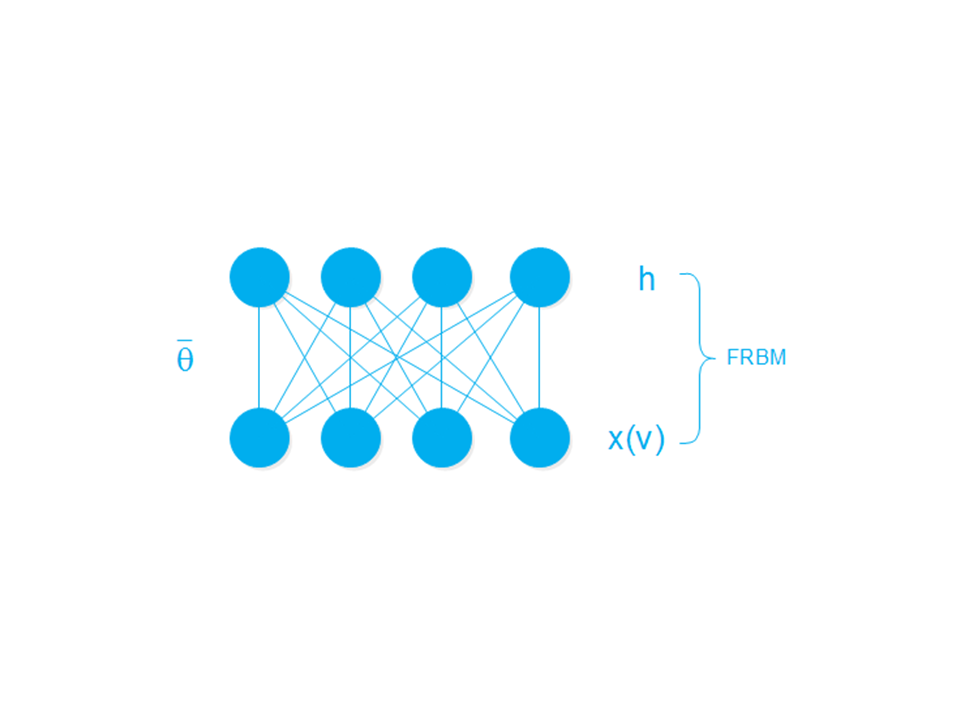

Supplement: S2 Fig — (ZIP) [file pone.0190831.s002.zip › S2 Fig/S2_Fig.tif]

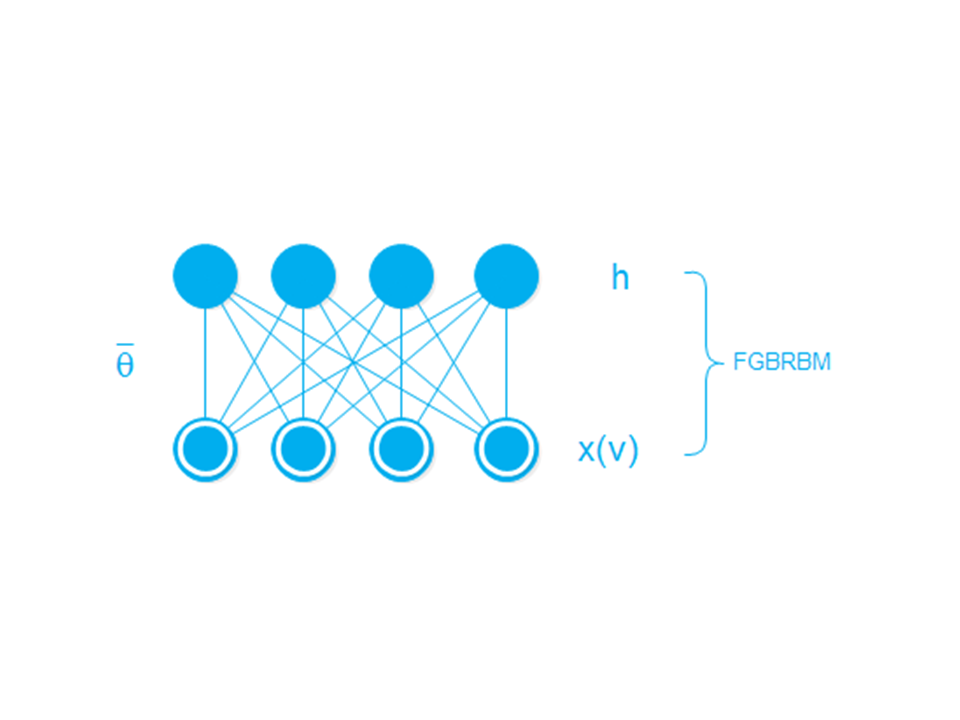

Supplement: S3 Fig — (ZIP) [file pone.0190831.s003.zip › S3 Fig/S3_Fig.tif]

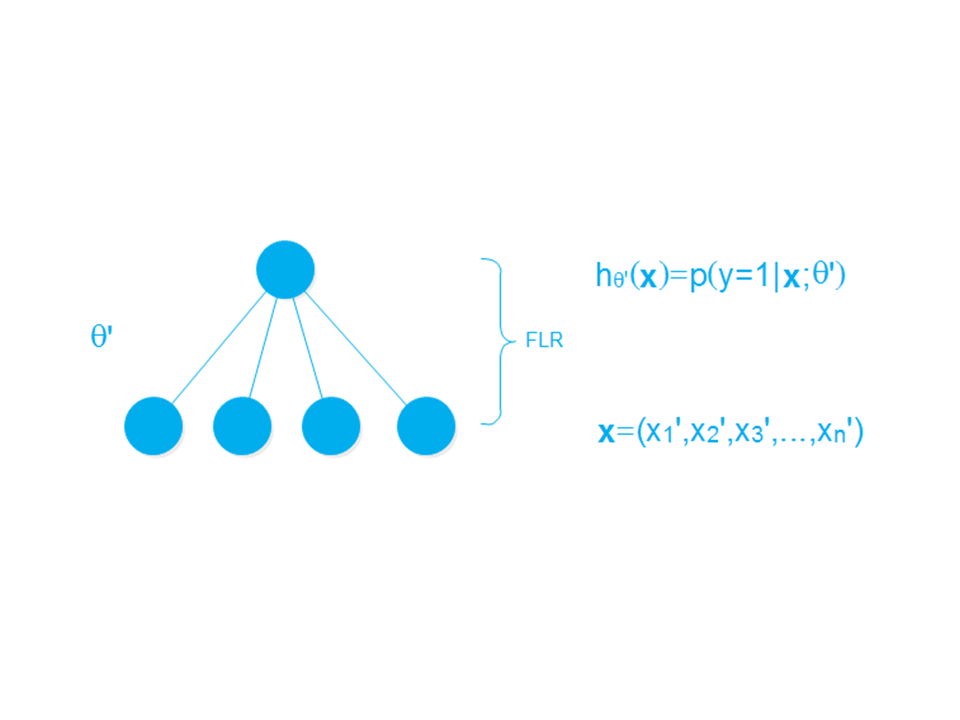

Supplement: S4 Fig — (ZIP) [file pone.0190831.s004.zip › S4 Fig/S4_Fig.tif]

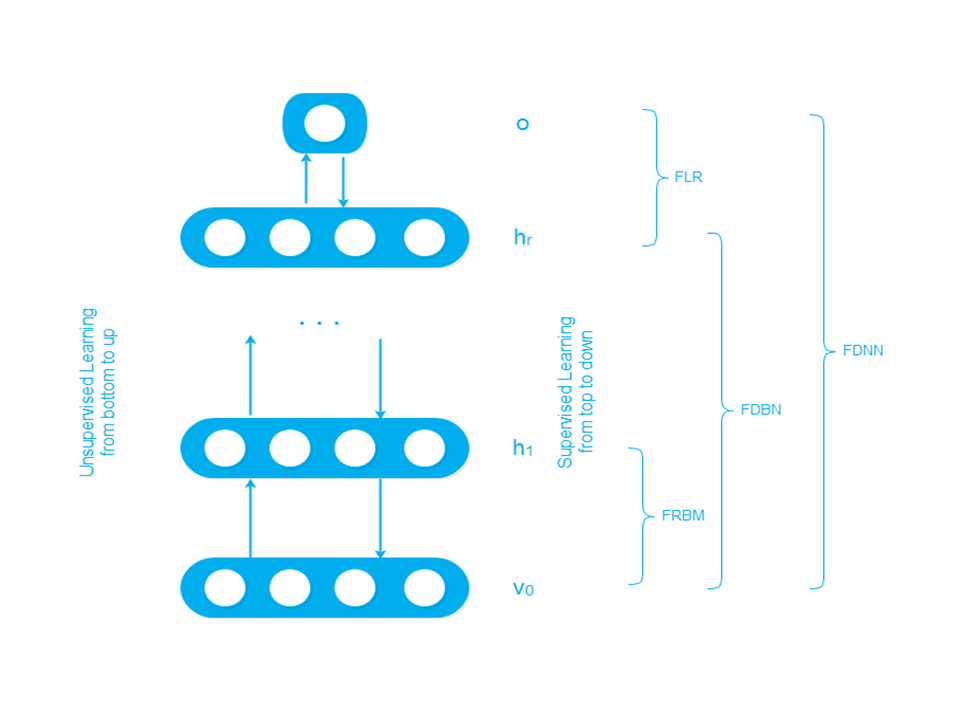

Supplement: S5 Fig — (ZIP) [file pone.0190831.s005.zip › S5 Fig/S5_Fig.tif]

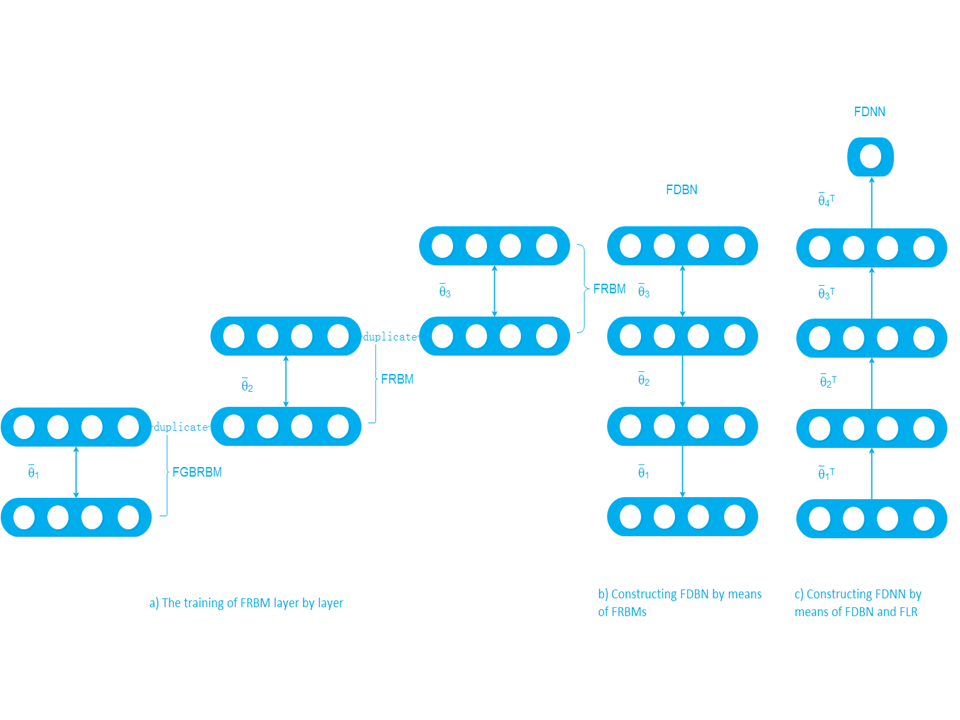

Supplement: S6 Fig — (ZIP) [file pone.0190831.s006.zip › S6 Fig/S6_Fig.tif]

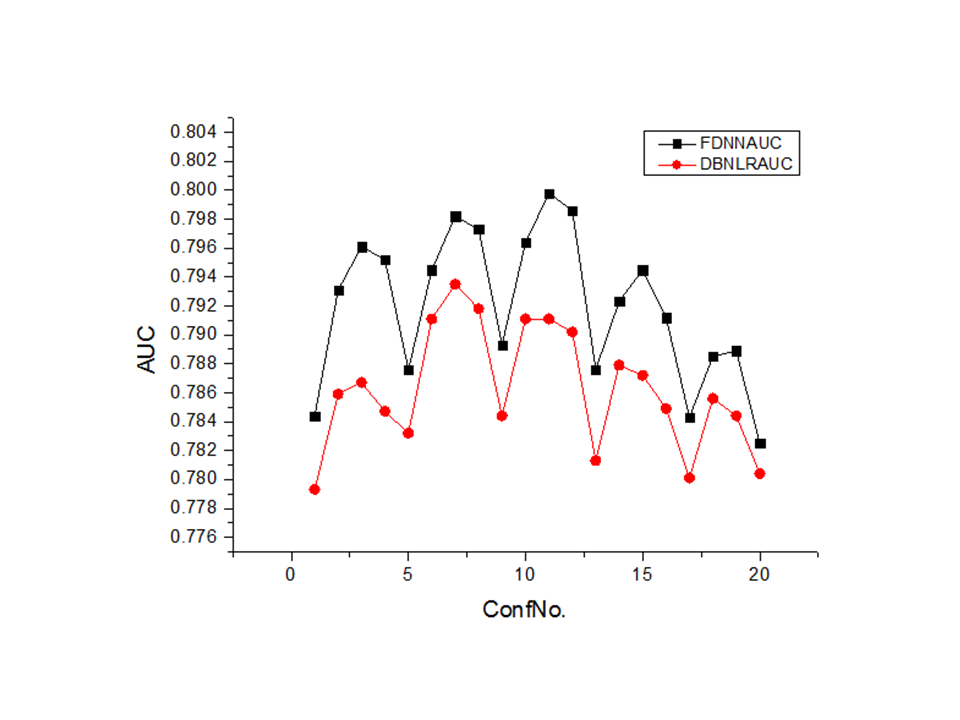

Supplement: S7 Fig — (ZIP) [file pone.0190831.s007.zip › S7 Fig/S7_Fig.tif]

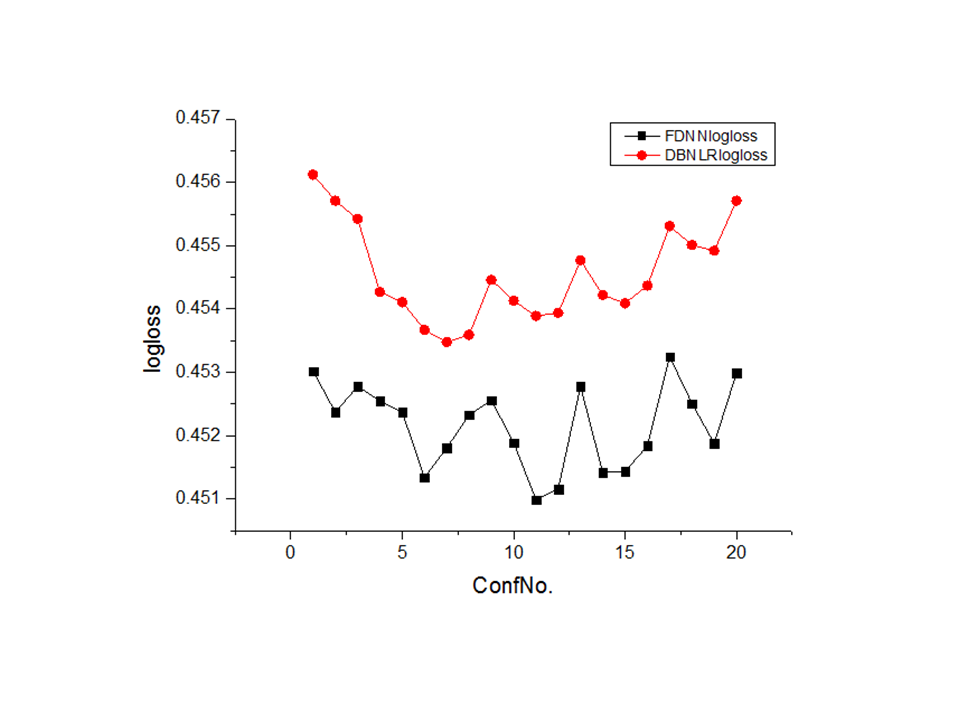

Supplement: S8 Fig — (ZIP) [file pone.0190831.s008.zip › S8 Fig/S8_Fig.tif]

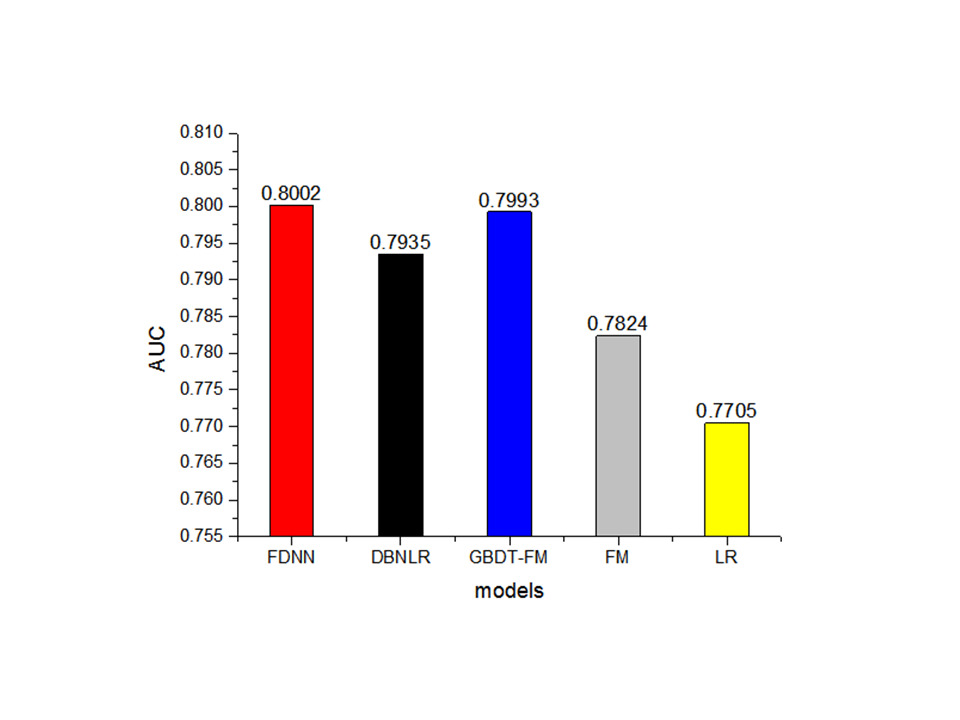

Supplement: S9 Fig — (ZIP) [file pone.0190831.s009.zip › S9 Fig/S9_Fig.tif]

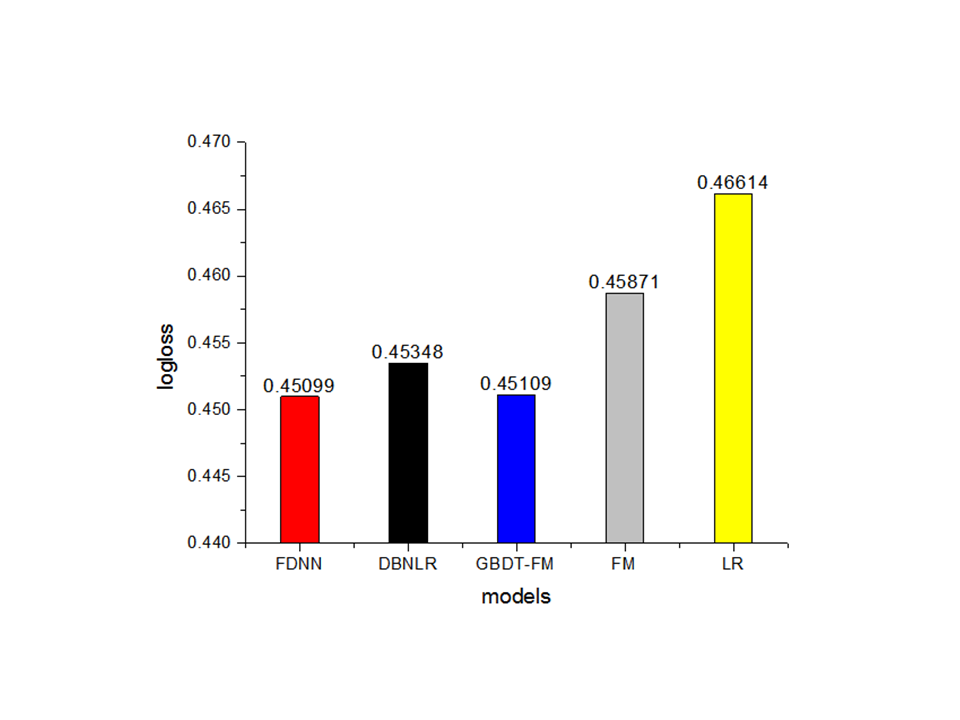

Supplement: S10 Fig — (ZIP) [file pone.0190831.s010.zip › S10 Fig/S10_Fig.tif]

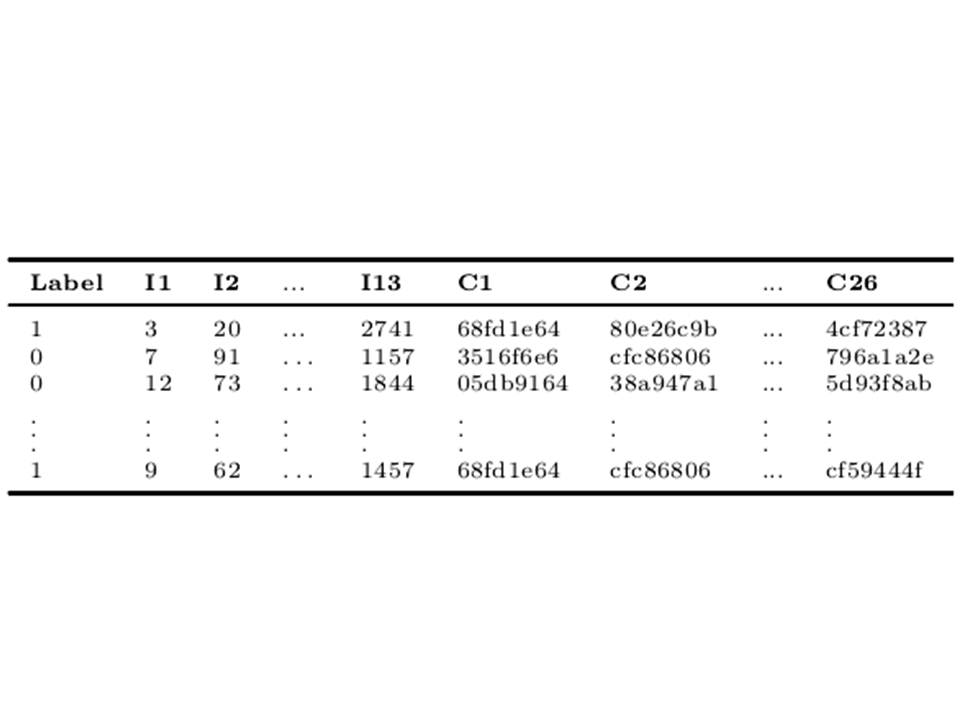

Supplement: S1 Table — (ZIP) [file pone.0190831.s011.zip › S1 Table/S1_Table.tif]

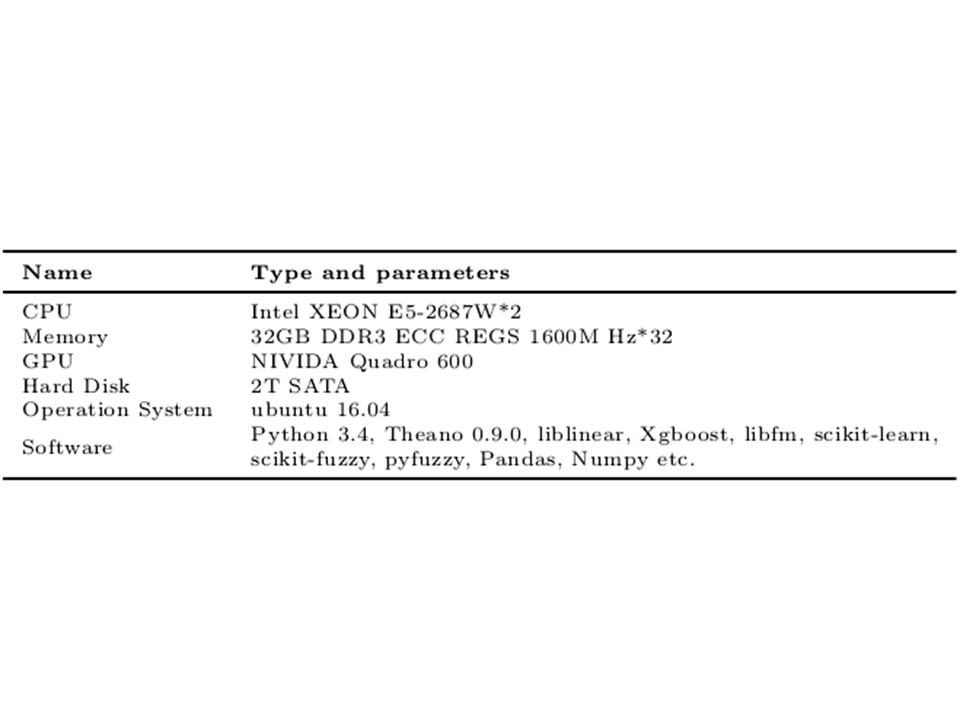

Supplement: S2 Table — (ZIP) [file pone.0190831.s012.zip › S2 Table/S2_Table.tif]

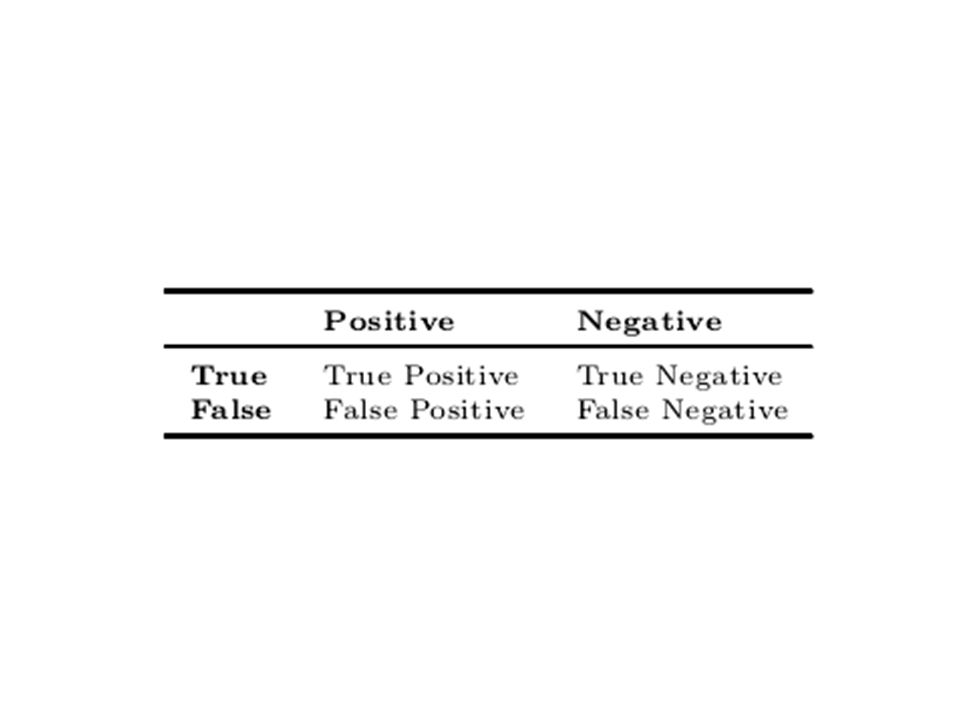

Supplement: S3 Table — (ZIP) [file pone.0190831.s013.zip › S3 Table/S3_Table.tif]

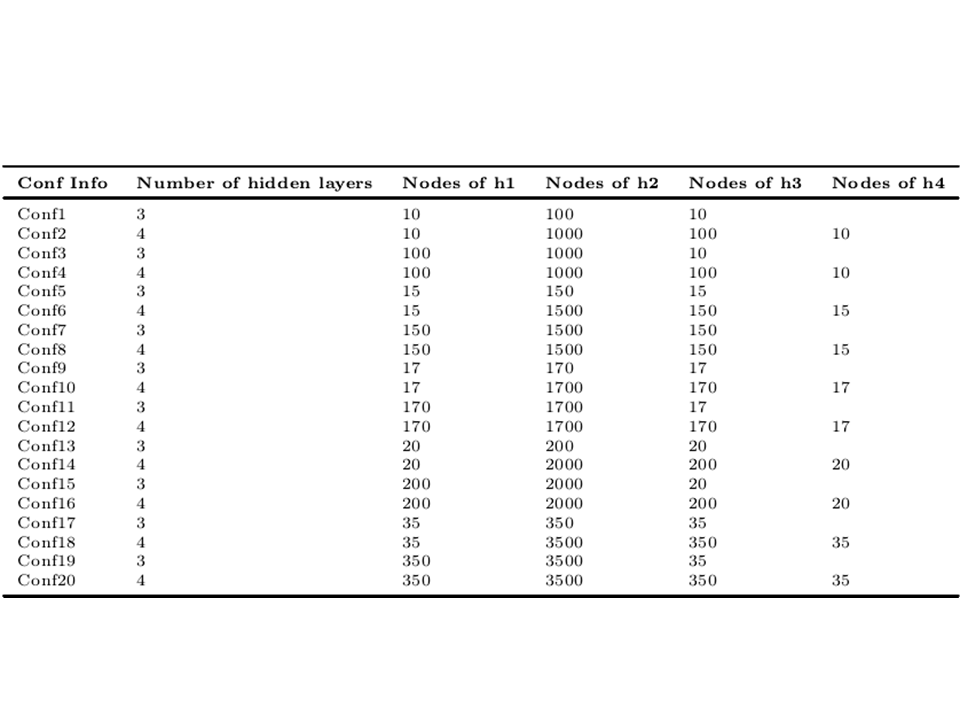

Supplement: S4 Table — (ZIP) [file pone.0190831.s014.zip › S4 Table/S4_Table.tif]
